# Collaborative update of a rule-based expert system for HIV-1 genotypic resistance test interpretation

**DOI:** 10.1371/journal.pone.0181357

**Published:** 2017-07-28

**Authors:** Roger Paredes, Philip L. Tzou, Gert van Zyl, Geoff Barrow, Ricardo Camacho, Sergio Carmona, Philip M. Grant, Ravindra K. Gupta, Raph L. Hamers, P. Richard Harrigan, Michael R. Jordan, Rami Kantor, David A. Katzenstein, Daniel R. Kuritzkes, Frank Maldarelli, Dan Otelea, Carole L. Wallis, Jonathan M. Schapiro, Robert W. Shafer

**Affiliations:** 1 IrsiCaixa AIDS Research Institute, Badalona, Spain; 2 Division of Infectious Diseases, Stanford University, Stanford, CA, United States of America; 3 Division of Medical Virology, Stellenbosch University and NHLS Tygerberg, Cape Town, South Africa; 4 Centre for HIV/AIDS Research, Education and Services (CHARES), Department of Medicine, University of the West Indies, Kingston Jamaica; 5 Rega Institute for Medical Research, Katholieke Universiteit Leuven, Leuven, Belgium; 6 Department of Molecular Medicine and Haematology, University of the Witwatersrand, Johannesburg, South Africa; 7 UCL, Department of Infection, London, WC1E 6BT, United Kingdom; 8 Amsterdam Institute for Global Health and Development, Department of Global Health, Academic Medical Center of the University of Amsterdam, Amsterdam, The Netherlands; 9 BC Centre for Excellence in HIV/AIDS, Vancouver, British Columbia, Canada; 10 Tufts University School of Medicine, Boston, MA, United States of America; 11 Division of Infectious Diseases, Alpert Medical School, Brown University, Providence, RI, United States of America; 12 Division of Infectious Diseases, Brigham and Women’s Hospital, Harvard Medical School, Boston, MA, United States of America; 13 HIV Dynamics and Replication Program, CCR, National Cancer Institute, NIH, Translational Research Unit, Frederick, MD, United States of America; 14 Molecular Diagnostics Laboratory, National Institute for Infectious Diseases, Bucharest, Romania; 15 BARC-SA and Lancet Laboratories, Johannesburg, South Africa; 16 National Hemophilia Center, Tel Hashomer, Israel; Instituto de Salud Carlos III, SPAIN

## Abstract

**Introduction:**

HIV-1 genotypic resistance test (GRT) interpretation systems (IS) require updates as new studies on HIV-1 drug resistance are published and as treatment guidelines evolve.

**Methods:**

An expert panel was created to provide recommendations for the update of the Stanford HIV Drug Resistance Database (HIVDB) GRT-IS. The panel was polled on the ARVs to be included in a GRT report, and the drug-resistance interpretations associated with 160 drug-resistance mutation (DRM) pattern-ARV combinations. The DRM pattern-ARV combinations included 52 nucleoside RT inhibitor (NRTI) DRM pattern-ARV combinations (13 patterns x 4 NRTIs), 27 nonnucleoside RT inhibitor (NNRTI) DRM pattern-ARV combinations (9 patterns x 3 NNRTIs), 39 protease inhibitor (PI) DRM pattern-ARV combinations (13 patterns x 3 PIs) and 42 integrase strand transfer inhibitor (INSTI) DRM pattern-ARV combinations (14 patterns x 3 INSTIs).

**Results:**

There was universal agreement that a GRT report should include the NRTIs lamivudine, abacavir, zidovudine, emtricitabine, and tenofovir disoproxil fumarate; the NNRTIs efavirenz, etravirine, nevirapine, and rilpivirine; the PIs atazanavir/r, darunavir/r, and lopinavir/r (with “/r” indicating pharmacological boosting with ritonavir or cobicistat); and the INSTIs dolutegravir, elvitegravir, and raltegravir. There was a range of opinion as to whether the NRTIs stavudine and didanosine and the PIs nelfinavir, indinavir/r, saquinavir/r, fosamprenavir/r, and tipranavir/r should be included. The expert panel members provided highly concordant DRM pattern-ARV interpretations with only 6% of NRTI, 6% of NNRTI, 5% of PI, and 3% of INSTI individual expert interpretations differing from the expert panel median by more than one resistance level. The expert panel median differed from the HIVDB 7.0 GRT-IS for 20 (12.5%) of the 160 DRM pattern-ARV combinations including 12 NRTI, two NNRTI, and six INSTI pattern-ARV combinations. Eighteen of these differences were updated in HIVDB 8.1 GRT-IS to reflect the expert panel median. Additionally, HIVDB users are now provided with the option to exclude those ARVs not considered to be universally required.

**Conclusions:**

The HIVDB GRT-IS was updated through a collaborative process to reflect changes in HIV drug resistance knowledge, treatment guidelines, and expert opinion. Such a process broadens consensus among experts and identifies areas requiring further study.

## Introduction

HIV-1 drug resistance is one of the main obstacles to the long-term effectiveness of antiretroviral (ARV) therapy. In upper-income countries, genotypic resistance testing (GRT) is performed routinely at diagnosis, treatment initiation, and at the time of virological failure (VF). In lower- and middle-income countries, it is performed in the public health sectors for *ad hoc* drug resistance surveillance and, increasingly, for managing patients with VF. Interpreting GRT results is one of the most difficult challenges facing HIV care providers because there are many drug-resistance mutations (DRMs) associated with each of the ARV classes. These DRMs have variable effects on *in vitro* ARV susceptibility and occur in many different combinations.

Because of the complexity inherent in GRT interpretation, automated interpretation systems have been developed to infer the extent of ARV resistance from DRMs in the targets of ARV therapy [1, 2]. The Stanford HIV Drug Resistance Database (HIVDB) GRT interpretation system (GRT-IS) is a rule-based system in which penalties are assigned to DRMs and to DRM combinations for ARVs in the four most commonly used ARV classes [3]: nucleoside RT inhibitors (NRTIs), nonnucleoside RT inhibitors (NNRTIs), protease inhibitors (PIs), and integrase strand transfer inhibitors (INSTIs). The resistance interpretation is determined by adding the DRM penalties for each ARV. The HIVDB GRT-IS also provides comments about each DRM in a submitted HIV-1 sequence.

The HIVDB GRT-IS DRM includes penalty scores and comments based on several types of data including the relative frequency of a DRM in ARV-naïve and ARV-experienced individuals; the contribution of the DRM to reduced *in vitro* susceptibility; and the association of the DRM with reduced virological response to an ARV regimen. This system requires updates as new studies on HIV drug resistance are published and as treatment guidelines evolve. Three of the authors of this study (RP, JMS, and RWS) organized a group of international experts to assist with updating the HIVDB GRT system. These experts, who regularly attend HIV drug resistance meetings and have published one or more peer-reviewed papers on HIV drug resistance, were polled on a variety of aspects of GRT interpretation including the analysis of specific DRM patterns. This manuscript describes the HIVDB GRT-IS and summarizes the authors’ opinions on some of the most relevant clinical topics in HIV GRT interpretation in light of recent publications and publicly available in vitro susceptibility data.

## Methods

### HIVDB genotypic resistance test (GRT) interpretation system (IS)

The HIVDB GRT is a rules-based system in which the resistance interpretation for 22 ARVs (Table 1) is determined by adding the ARV penalties for each of the DRMs present in a virus sample. A total penalty score of <10 indicates susceptibility; 10 to 14 indicates potential low-level resistance; 15 to 29 indicates low-level resistance; 30 to 59 indicates intermediate resistance; and ≥60 indicates high-level resistance. Mutation penalties are assigned both to individual DRMs and to combinations of DRMs.

**Table 1:** List of antiretroviral (ARV) drugs and their abbreviations by ARV class.

ARV Class	Abbreviation	Generic Name
Nucleoside RT Inhibitors (NRTIs)	3TC	Lamivudine
	ABC	Abacavir
	AZT	Zidovudine
	D4T	Stavudine
	DDI	Didanosine
	FTC	Emtricitabine
	TDF	Tenofovir disoproxil fumarate
Nonnucleoside RT Inhibitors (NNRTIs)	EFV	Efavirenz
	ETR	Etravirine
	NVP	Nevirapine
	RPV	Rilpivirine
Protease Inhibitors (PIs)*	ATV	Atazanavir
	DRV	Darunavir
	FPV	Fosamprenavir
	IDV	Indinavir
	LPV	Lopinavir
	NFV	Nelfinavir
	SQV	Saquinavir
	TPV	Tipranavir
Integrase Strand-Transfer Inhibitors (INSTIs)	DTG	Dolutegravir
	EVG	Elvitegravir
	RAL	Raltegravir

*With the exception of NFV, each of the PIs are usually administered with a drug to boost PI levels. Ritonavir, usually indicated by “/r” is available for ATV, DRV, FPV, IDV, LPV, SQV, and TPV. Cobicistat, usually indicated by “/c” or “/cobi” is available for ATV and DRV.

The HIVDB GRT-IS classification "Susceptible" is assigned when a virus displays no evidence reduced susceptibility when compared with a wild-type virus. "Potential low-level resistance" is assigned when a virus has DRMs consistent with previous ARV exposure or contains DRMs associated with resistance only when they occur with other DRMs. "Low-level resistance" is assigned when a virus has DRMs associated with reduced *in vitro* ARV susceptibility or a suboptimal virological response to ARV treatment. "Intermediate resistance" is assigned when, although there is a high likelihood that an ARV’s activity would be reduced in the presence of a virus’s DRMs, the ARV would likely still retain significant antiviral activity against the virus. "High-level resistance" is assigned when a virus has DRMs predicted to confer a level of resistance similar to that observed in viruses with the highest levels of reduced *in vitro* susceptibility or in viruses that have little or no virological response to ARV treatment [3].

In June 2016, the expert panel was provided access to the five sources of background material outlined in Table 2: (i) DRM penalty scores for HIVDB version 7.0 (February 2014) and an interim update (version 8.0; June 2016); (ii) A list of the calculated summed version 8.0 DRM penalty scores for each of the distinct DRM patterns present in sequences in HIVDB; (iii) Mutation classifications updated in June 2016; (iv) Mutation comments updated in June 2016; and (v) Mutation notes updated June 2016. These five sources of background material were compiled by RWS and reviewed by RP and JMS to extract specific material to be evaluated by the expert panel.

**Table 2:** Materials available for review by the expert panel.

Material and Description	Link
**Mutation Scores:** For each ARV class, a table contained lists of individual DRMs and their associated scores, followed by a list of DRM combinations and their associated scores. Mutation penalty scores were multiples of 5 and ranged from -15 (increased ARV activity) to 60 (loss of ARV activity). ARV activity was estimated by adding the penalties for each DRM in a sequence and converting the total score to one of five interpretations: (i) “Susceptible”, total score <10; (ii) “Potential low-level resistance”, total score between 10 and 14; (iii) “Low-level resistance”, total score between 15 and 29; (iv) “Intermediate resistance”, total score between 30 and 59; and (v) “High-level resistance”, total score ≥60.	• https://hivdb.stanford.edu/dr-summary/mut-scores/NRTI/ • https://hivdb.stanford.edu/dr-summary/mut-scores/NNRTI/ • https://hivdb.stanford.edu/dr-summary/mut-scores/PI/ • https://hivdb.stanford.edu/dr-summary/mut-scores/INSTI/
**Mutation Pattern Scores:** A list of the calculated summed DRM penalty scores for each of the distinct DRM patterns present in HIVDB sequences.	• https://hivdb.stanford.edu/dr-summary/pattern-scores/NRTI/ • https://hivdb.stanford.edu/dr-summary/pattern-scores/NNRTI/ • https://hivdb.stanford.edu/dr-summary/pattern-scores/PI/ • https://hivdb.stanford.edu/dr-summary/pattern-scores/INSTI/
**Mutation Classifications:** (i) PR mutations were classified as “Major”, “Accessory”, or “Other; (ii) RT mutations were classified as “NRTI”, ‘NNRTI”, or “Other”; and (iii) IN Mutations were classified as “Major”, “Accessory”, or “Other”.	In the comments sections (below)
**Mutation Comments:** For each ARV class, there was a comment for each mutation with a penalty score and for several mutations without mutation penalty scores, which were once considered to be associated resistance.	• https://hivdb.stanford.edu/dr-summary/comments/NRTI/ • https://hivdb.stanford.edu/dr-summary/comments/NNRTI/ • https://hivdb.stanford.edu/dr-summary/comments/PI/ • https://hivdb.stanford.edu/dr-summary/comments/INSTI/
**Mutation Notes:** For each ARV class, there was an HTML page containing a summary of the DRMs associated with that class.	• https://hivdb.stanford.edu/dr-summary/resistance-notes/NRTI/ • https://hivdb.stanford.edu/dr-summary/resistance-notes/NNRTI/ • https://hivdb.stanford.edu/dr-summary/resistance-notes/PI/ • https://hivdb.stanford.edu/dr-summary/resistance-notes/INSTI/

ARV: antiretroviral; DRM: drug resistance mutation; NRTI: nucleoside RT inhibitor; NNRTI: nonnucleoside RT inhibitor; PI: protease inhibitor; INSTI: integrase strand transfer inhibitor

The expert panel was asked to provide feedback on the following aspects of GRT interpretation: (i) The ARVs and the extent to which pharmacologic considerations should be included in a GRT report; (ii) The predicted drug resistance levels associated with 52 NRTI DRM pattern-ARV combinations (13 patterns x 4 NRTIs), 27 NNRTI DRM pattern-ARV combinations (9 patterns x 3 NNRTIs), 39 PI DRM pattern-ARV combinations (13 patterns x 3 PIs) and 42 INSTI DRM pattern-ARV combinations (14 patterns x 3 INSTIs). Feedback was solicited through the distribution of blank worksheets (S1 File).

### DRM patterns: Selection and analysis

The DRM patterns were selected to reflect several challenges in GRT interpretation: (i) The extent to which the most commonly occurring TDF-associated DRMs are likely to interfere with the success of TDF-containing regimens; (ii) The clinical significance of thymidine analog mutations (TAMs) for TDF, ABC, and cytosine-analog (3TC and FTC)-containing regimens; (iii) The DRM patterns associated with high-level resistance to the ARVs with the highest genetic barrier to resistance (DTG and pharmacologically boosted DRV and LPV); and (iv) The extent to which certain polymorphic DRMs (i.e., DRMs that are selected by ARV therapy but that also occur in the absence of therapy) might interfere with success of NNRTI- and INSTI-containing regimens.

The expert panel members were asked to assign interpretations of susceptible, potential low-level resistance, low-level resistance, intermediate resistance, or high-level resistance to five NRTIs (3TC, ABC, AZT, FTC, and TDF), three NNRTIs (EFV, RPV, and ETR), three PIs (ATV/r, DRV/r, and LPV/r), and three INSTIs (DTG, EVG, and RAL). The cytosine analogs 3TC and FTC were considered by each expert to have similar drug-resistance interpretations and are henceforth referred to as 3FTC. ATV, DRV, and LPV were each considered to be pharmacologically boosted by either ritonavir or cobicistat.

Expert panel members were instructed to leave an ARV interpretation for a DRM pattern blank if they were uncertain of the pattern’s effect on that ARV. Each panel member’s scores were anonymous to all but RP, JMS, and RWS. After all interpretations were submitted, those who provided an outlier interpretation–defined as differing from the median interpretation by more than one level–were asked to review their interpretation to exclude the possibility that the submitted result was an error.

For each DRM pattern-ARV combination, consistency among panel members was assessed using the mean absolute deviation from the median expert level. All differences between HIVDB 7.0 and the median expert panel level were reviewed by RP, JMS, and RWS. The effects of proposed HIVDB scoring changes were evaluated by re-interpreting the complete set of distinct DRM patterns in HIVDB using the updated scores. This process generated tables for each ARV class that were identical in format to those on the four Mutation Pattern Scores pages (Table 2). DRM patterns influenced by a scoring change (i.e., a scoring change that resulted in a changed resistance level) were sorted by their frequency. The updated scoring system was called HIVDB version 8.1 because it replaced the interim version HIVDB 8.0 completed in June 2016. HIVDB 8.1 was released online September 19, 2016. Each of the HIVDB 8.1 scores and each of the changes between HIVDB 7.0 and 8.1 are available in the supplementary material and at https://hivdb.stanford.edu/page/version-updates/#version.8.1.1.update.2016-09-15.

### Relative ARV susceptibility profiles

For each ARV class, we tabulated the proportion of viruses in HIVDB with each distinct pattern of DRMs for that class. We then determined the HIVDB 8.1 resistance interpretation for the NRTIs 3TC, ABC, AZT, FTC, and TDF for each NRTI DRM pattern; for the NNRTIs EFV, ETR, and RPV for each NNRTI DRM pattern; for the PIs ATV/r, DRV/r, and LPV/r for each PI DRM pattern; and for the INSTIs DTG, EVG, and RAL for each INSTI DRM pattern.

Sequences with DRM patterns resulting in similar profiles of relative ARV susceptibility were pooled to determine the frequency of each distinct cross-resistance profile within each ARV class. To reduce the number of potential relative susceptibility profiles (i.e. number ARVs ^number of ARV levels^) from 5^4^ (625) for the NRTIs and 5^3^ (125) for the NNRTIs, PIs and INSTIs to a more manageable number, we pooled viruses with potential low-level and low-level susceptibility which resulted in 4^4^ (256) potential profiles for the NRTIs and 4^3^ (64) potential profiles for the NNRTIs, PIs, and INSTIs.

The most common relative ARV susceptibility profiles are listed in separate tables for each ARV class in which each profile is also associated with the three most common DRM patterns responsible for the profile.

### *In vitro* susceptibility (phenotype) data

Following the expert panel’s assessments, we created tabular summaries of phenotypic data from HIVDB for each of the NRTI, NNRTI, PI, and INSTI DRM patterns selected for analysis. Each tabular summary contained the DRM pattern, the proportion of viruses in HIVDB that exactly matched the DRM pattern, and the median fold reduction in susceptibility produced by the pattern for each ARV. Exactly matching DRM patterns were defined as not having additional major DRMs. For the NRTIs, these included DRMs at RT positions 41, 65, 67, 70, 74, 115, 184, 210, and 215. For the NNRTIs, these included DRMs at RT positions 100, 101, 103, 106, 181, 188, 190, and 230. For the PIs, these included DRMs at protease positions 30, 32, 46, 47, 48, 50, 54, 76, 82, 84, 88, and 90. For the INSTIs, these included DRMs at integrase positions 66, 92, 118, 121, 138, 140, 143, 147, 148, 155, and 263.

For the NRTI, NNRTI, and PI classes, the tables contained phenotypic data determined by the PhenoSense assay [4, 5]. Because fewer phenotypic data were available for the INSTI class, the INSTI table contained susceptibility data determined by the PhenoSense assay and by the ViiV HeLa-CD4 reporter gene assay, which for the INSTIs provides results similar to the PhenoSense assay [6]. Virus isolates containing electrophoretic mixtures at DRM positions were excluded. For composite DRM patterns, separate rows of data were provided for each individual pattern. For example, for the NRTI DRM pattern “T215YF”, there were separate rows for “T215Y” and “T215F”. The number of phenotypic tests for a pattern was indicated by a subscript following the median fold reduction in susceptibility.

Fold-reductions in susceptibility considered to be associated with partial loss of clinical activity were indicated in bold whereas those considered to be associated with near-complete loss of clinical activity were indicated in bold and underlined. Such ARV-specific “clinical cut-offs” are commonly used because the clinical significance of different levels of reduction in susceptibility differs among ARVs. These cut-offs, however, should be considered rough guides of expected clinical activity for the following reasons. First, these cut-offs have usually been derived from the retrospective analysis of just a single clinical trial. Second, for some ARVs, there is only one cut-off (rather than separate low and high cut-offs). Finally, for other ARVs, there is only a “biological” cut-off, designed to distinguish reduced susceptibility from the naturally occurring variation in susceptibility observed in viruses from ARV-naïve persons lacking DRMs [7]. In this paper, we define two cut-offs for each ARV relying primarily on published studies, the Monogram Biosciences template report, and in some cases by extrapolating from closely related ARVs [5, 8–13].

## Results

### ARVs to include in a GRT interpretation system

Each of the 16 panel members recommended reporting susceptibility estimates for each of the four NNRTIs and three INSTIs currently reported by HIVDB 7.0. Each also recommended reporting susceptibility for the five NRTIs: 3FTC, ABC, AZT, and TDF. However, just 11 and five members, respectively, recommended reporting susceptibility estimates for d4T and ddI, which are no longer recommended for routine use. Each of the panel members recommended reporting results for ATV/r, DRV/r, and LPV/r. However, just 12, nine, seven, three, and one panel member recommended reporting results for TPV/r, FPV/r, SQV/r, IDV/r, and NFV, respectively.

The panel members each recommended that PI interpretations should be for boosted PIs. Six panel members recommended that the type of PI boosting (ritonavir vs. cobicistat) should be indicated even though it would not affect susceptibility estimates. Eleven panel members recommended indicating when DRV/r and DTG should be administered at the higher of their two recommended dosages [12, 14]. Three panel members advised distinguishing between TDF and TAF but no panel member recommended different susceptibility estimates for the two tenofovir prodrugs.

### Effects of DRM patterns on predicted NRTI susceptibility

**Fig 1:**
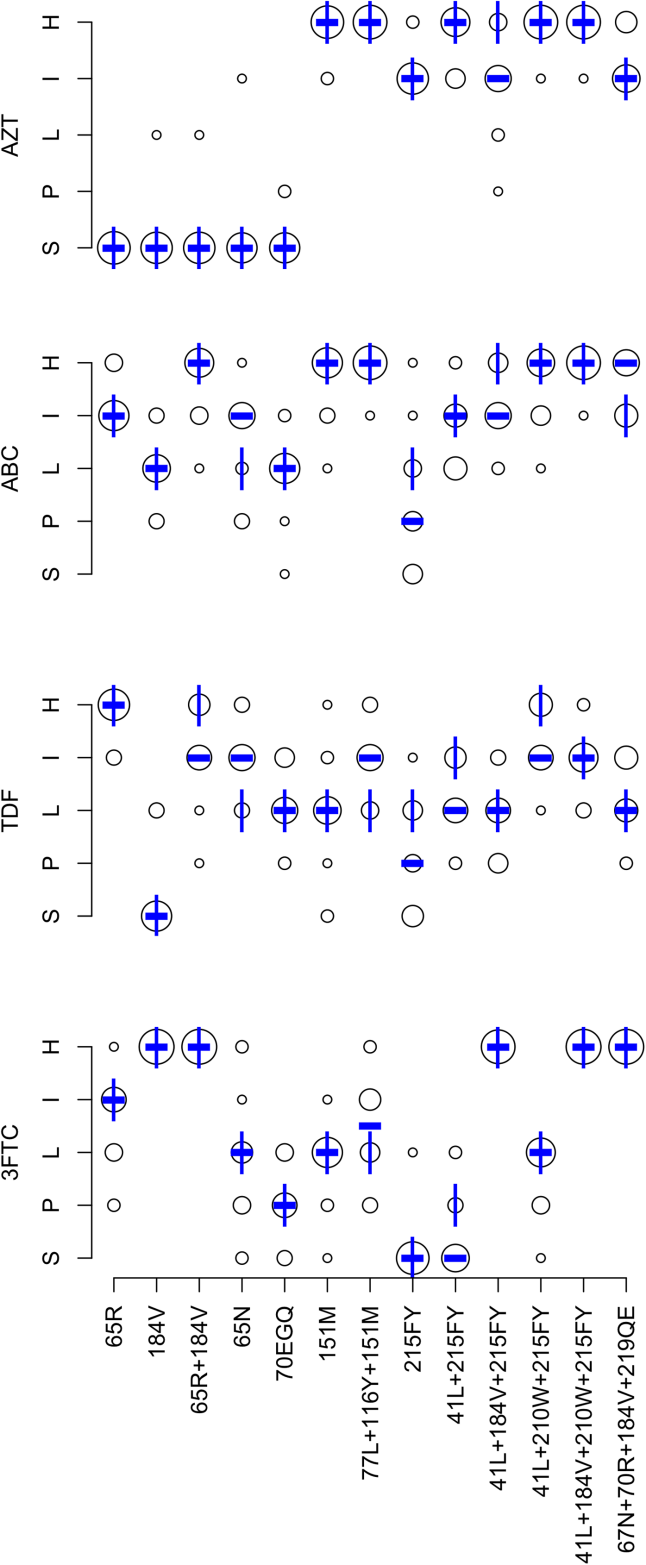
Expert panel assessments of 14 NRTI-associated drug-resistance mutation (DRM) patterns. Abbreviations: ABC (abacavir), AZT (zidovudine), TDF (tenofovir), 3FTC (lamivudine and emtricitabine), S (susceptible), P (potential low-level resistance), L (low-level resistance), I (intermediate resistance), H (high-level resistance). The diameter of each circle is proportional to the number of experts at the assigned level shown on the Y-axis. The bold dash is the median of the expert assessments. The vertical lines represent the HIVDB version 7.0 interpretations.

Fig 1 summarizes the expert panel’s NRTI interpretations. The completion rate was 97.4% for the 832 DRM pattern-ARV-interpretations (13 patterns x 4 NRTIs x 16 experts). Originally, 46 (5.5%) pattern-ARV-interpretations differed from the expert median by more than one-level. Following re-evaluation of outliers, 32 (3.8%) pattern-ARV-interpretations differed by more than one level.

Overall, the mean absolute deviation from the expert panel median was 0.34 levels: <0.5 levels for 38 pattern-ARV combinations and 0.5 to 1.0 for 14 pattern-ARV combinations. There was a one-level difference between the expert median and HIVDB 7.0 for 12 of 52 pattern-ARV combinations: six for TDF, four for ABC, one for 3FTC, and one for AZT. Table 3 lists these differences and indicates that for each a change was made to the HIVDB scoring system such that HIVDB 8.1 yielded a result that matched the expert median.

**Table 3:** Comparison of the HIVDB version 7.0, expert panel median, and eventual HIVDB version 8.1 levels for the 20 DRM pattern / ARV combinations with a difference between HIVDB version 7.0 and the expert panel median.

Class	ARV	Pattern	Version 7.0	Panel Median	Version 8.1
NRTI	TDF	65R+184V	High	Intermediate	Intermediate
		65N	Low	Intermediate	Intermediate
		77L+116Y+151M	Low	Intermediate	Intermediate
		215FY	Low	Potential	Potential
		41L+215FY	Intermediate	Low	Low
		41L+210W+215FY	High	Intermediate	Intermediate
	ABC	65N	Low	Intermediate	Intermediate
		215FY	Low	Potential	Potential
		41L+184V+215FY	High	Intermediate	Intermediate
		67N+70R+184V+219QE	Intermediate	High	High
	3FTC	41L+215FY	Potential	Susceptible	Susceptible
	AZT	41L+184V+215Y	High	Intermediate	Intermediate
NNRTI	EFV	98G	Potential	Low	Low
	ETR	138A	Susceptible	Potential	Potential
INSTI	RAL	97A	Low	Potential	Potential
		157Q	Low	Potential	Potential
		118R	Intermediate	Low	No change
	EVG	157Q	Low	Potential	Potential
		118R	Intermediate	Low	No change
	DTG	118R	Potential	Low	Low

After updating the scoring system, we determined the relative NRTI susceptibility profiles of 35,377 viruses in HIVDB with 4,749 distinct NRTI DRM patterns. Of the 256 possible profiles, 21 occurred in at least 1% of published sequences. Table 4 displays these 21 profiles, which accounted for 93.3% of viruses with one or more NRTI DRM. The most common relatively susceptibility profile–susceptibility to AZT and TDF, low-level resistance to ABC, and high-level resistance to 3FTC–occurred in 23.7% of all viruses with one or more NRTI DRMs. This profile was usually caused by M184V/I alone or M184V/I in combination with a second DRM with low mutation penalty scores.

**Table 4:** Patterns of predicted relative NRTI susceptibility profiles for 35,377 viruses in HIVDB with 4,749 Distinct NRTI-Resistance patterns*.

AZT	ABC	XTC	TDF	% of Total Relative NRTI Susceptibility Profiles^†^	Example DRM Patterns^§^	DRM Pattern % with Profile	DRM Pattern % of Total
Susc	Low	High	Susc	23.7	M184V	80.4	19.0
					A62V,M184V	3.7	0.9
					M41L,M184V	3.4	0.8
High	High	High	Int	10.2	M41L,M184V,L210W,T215Y	14.5	1.5
					D67N,K70R,M184V,T215F,K219Q	6.1	0.6
					M41L,D67N,M184V,L210W,T215Y	5.5	0.6
High	High	High	High	8.0	M41L,E44D,D67N,M184V,L210W,T215Y	6.2	0.5
					M41L,E44D,D67N,T69D,M184V,L210W,T215Y	5.0	0.4
					M41L,E44D,D67N,M184V,L210W,T215Y,K219N	2.7	0.2
High	High	Low	High	6.0	M41L,D67N,L210W,T215Y	11.0	0.7
					M41L,E44D,D67N,L210W,T215Y	6.2	0.4
					M41L,D67N,K70R,T215F,K219Q	4.3	0.3
Low	Susc	Susc	Susc	4.7	M41L	19.9	0.9
					T215S	19.4	0.9
					T215D	13.0	0.6
Int	High	High	Low	4.1	D67N,K70R,M184V,K219Q	43.2	1.8
					M41L,L74V,M184V,T215Y	10.2	0.4
					D67N,K70R,M184V,K219E	9.6	0.4
High	Int	Susc	Int	3.9	D67N,K70R,T215F,K219Q	14.4	0.6
					M41L,D67N,T215Y	6.9	0.3
					M41L,L210W,T215D	5.5	0.2
Int	Int	High	Low	3.5	M41L,M184V,T215Y	61.2	2.1
					M41L,M184V,T215F	13.0	0.5
					M41L,D67N,M184V,L210W	5.5	0.2
Susc	Susc	Susc	Susc	3.4	A62V	49.3	1.7
					K219Q	8.8	0.3
					F77L	5.8	0.2
Int	Low	High	Susc	2.9	M184V,T215Y	39.7	1.1
					D67N,K70R,M184V	21.2	0.6
					M184V,T215F	17.3	0.5
Int	Susc	Susc	Susc	2.7	K70R	33.2	0.9
					T215Y	29.5	0.8
					D67N,K70R	10.3	0.3
Int	Low	Susc	Low	2.4	M41L,T215D	15.3	0.4
					M41L,T215S	11.7	0.3
					M41L,T215E	8.3	0.2
Susc	High	High	Susc	2.2	L74V,M184V	40.2	0.9
					L74V,Y115F,M184V	15.5	0.3
					L74I,M184V	9.7	0.2
Susc	High	High	Int	2.0	K65R,M184V	47.7	1.0
					A62V,K65R,M184V	14.0	0.3
					K65R,M184I	8.9	0.2
High	High	High	Low	1.9	D67N,K70R,M184V,T215I,K219E	14.6	0.3
					D67N,K70R,M184V,T215V,K219Q	9.9	0.2
					D67N,K70R,M184V,T215I,K219Q	8.8	0.2
High	Low	Susc	Low	1.7	D67N,K70R,K219Q	39.5	0.7
					L210W,T215Y	9.6	0.2
					D67N,K70R,K219E	6.5	0.1
Low	Low	High	Susc	1.6	K70R,M184V	76.6	1.3
					M41L,D67N,M184V	4.0	0.1
					D67N,M184V,K219Q	3.1	0.1
High	Int	Susc	Low	1.5	M41L,T215Y	77.0	1.2
					M41L,T215F	15.0	0.2
					D67N,K70R,L74V,K219Q	2.2	0.0
High	Int	High	Low	1.5	M41L,D67N,M184V,T215Y	19.7	0.3
					M41L,D67N,T69D,M184V,T215Y	5.4	0.1
					M41L,A62V,M184V,T215Y	4.6	0.1
High	High	Low	Int	1.5	M41L,L210W,T215Y	71.9	1.0
					M41L,L74V,L210W,T215Y	13.2	0.2
					M41L,L210W,T215F	3.3	0.0
High	High	Int	High	1.3	M41L,D67N,K70R,L210W,T215Y,K219E	3.2	0.0
					M41L,E44D,D67N,K70R,L210W,T215Y,K219E	2.5	0.0
					M41L,T69Insertion,L210W,T215Y	2.3	0.0
Int	Int	High	Susc	1.3	M41L,M184V,L210W	23.9	0.3
					D67N,M184V,T215Y	7.7	0.1
					A62V,M184V,T215Y	5.0	0.1
Susc	Int	Int	High	1.3	K65R	73.7	0.9
					A62V,K65R	4.2	0.1
					K65R,K219R	2.9	0.0

*Obtained from HIVDB (https://hivdb.stanford.edu/dr-summary/pattern-scores/INSTI/) January 2017. For the purposes of this analysis, the HIVDB interpretations of “Susceptible” and “Potential Low resistance” were grouped together as “Susceptible”.

^**†**^Relative susceptibility patterns accounting for ≥1% of all such patterns are shown. These patterns account for the HIVDB interpretations of 93.3% of sequences containing ≥1 NRTI DRM.

^**§**^For each relative NRTI susceptibility pattern, the three most common NRTI DRM patterns responsible for the relative susceptibility pattern are shown as examples.

Table 5 summarizes available phenotypic data in HIVDB for the 13 DRM patterns. HIVDB contained many phenotypic results for M184V alone, K65R alone, M184V+K65R, and for several of the patterns with multiple TAMs ± M184V. However, HIVDB contained few results for several other patterns including the TDF-associated DRMs K65N and K70E/Q/G [15].

**Table 5:** In vitro susceptibilities associated with the 13 NRTI drug resistance mutation (DRM) patterns.

Overall Pattern*	Specific Pattern*	Exact^†^	Included^†^	3TC^§^	ABC^§^	AZT^§^	TDF^§^
M184V	M184V	19.03%	63.33%	**>200**_**175**_	3.1_125_	0.5_124_	0.5_63_
K65R	K65R	0.93%	5.64%	**8.9**_**30**_	2.5_20_	0.5_20_	**1.8**_**17**_
K65R, M184V	K65R, M184V	0.96%	2.88%	**>200**_**27**_	**8.4**_**16**_	0.4_16_	1.2_16_
K65N	K65N	0.02%	0.10%	**7.3**_**1**_	2.1_1_	-	**1.7**_**1**_
K70EGQ	K70E	0.07%	0.85%	**5.3**_**5**_	1.4_3_	0.2_2_	0.9_3_
	K70G	0.00%	0.31%	-	-	-	-
	K70Q	0.03%	0.27%	-	-	-	-
Q151M	Q151M	0.03%	2.74%	**3**_**16**_	**5.5**_**10**_	**7**_**10**_	1_9_
F77L, F116Y, Q151M	F77L, F116Y, Q151M	0.01%	1.04%	**4.4**_**2**_	**6.8**_**2**_	**48**_**2**_	**1.6**_**2**_
T215YF	T215Y	0.80%	28.76%	2.4_19_	1.6_12_	**7.4**_**15**_	**1.4**_**14**_
	T215F	0.21%	10.29%	2.4_4_	1.8_2_	**5**_**2**_	1.3_2_
M41L, T215YF	M41L, T215Y	1.18%	23.87%	2_15_	2_9_	**12**_**12**_	1.3_7_
	M41L, T215F	0.23%	5.24%	2.6_1_	3.2_1_	**50**_**1**_	-
M41L, M184V, T215YF	M41L, M184V, T215Y	2.13%	14.34%	**>200**_**55**_	**5.1**_**41**_	**6**_**41**_	1.1_24_
	M41L, M184V, T215F	0.45%	3.39%	**>200**_**6**_	**5.4**_**7**_	**3.5**_**7**_	0.5_1_
M41L, L210W, T215YF	M41L, L210W, T215Y	1.05%	16.44%	2.8_34_	3.1_19_	**164**_**21**_	**3.1**_**18**_
	M41L, L210W, T215F	0.05%	1.04%	**3.1**_**4**_	3.2_1_	**217**_**3**_	**4.1**_**2**_
M41L, M184V, L210W, T215YF	M41L, M184V, L210W, T215Y	1.48%	9.65%	**>200**_**69**_	**6.5**_**48**_	**18**_**51**_	**1.6**_**38**_
	M41L, M184V, L210W, T215F	0.10%	0.66%	**148**_**1**_	-	**69**_**1**_	**2.8**_**1**_
D67N, K70R, M184V, K219QE	D67N, K70R, M184V, K219Q	1.76%	6.12%	**>200**_**12**_	**5.6**_**9**_	**4.6**_**9**_	**1.4**_**5**_
	D67N, K70R, M184V, K219E	0.39%	2.85%	**>200**_**4**_	3.9_3_	1.7_3_	0.7_2_

*Mutation patterns are defined as those matching the listed DRMs and not containing additional DRMs at positions 41, 65, 67, 70, 74, 115, 151, 184, 210, and 215.

^**†**^Exact: % of sequences in HIVDB exactly matching the mutation pattern; Included: % of sequences in HIVDB matching or including the mutation pattern.

^**§**^Median fold reduced susceptibility as determined by the PhenoSense assay (Monogram Biosciences, South San Francisco). Sequences with electrophoretic mixtures were excluded. “-”indicates that no phenotype results were available for a particular DRM pattern / NRTI combination. Fold reductions in susceptibility ≥1.4 fold for TDF, ≥2 fold AZT, ≥3 fold for 3TC, and ≥4.5 fold ABC are in bold consistent with the PhenoSense assay cut-off for partial loss of clinical efficacy [5]. Fold reductions in susceptibility ≥4 fold for TDF and ≥6.5 fold for ABC are also underlined consistent with the PhenoSense assay upper cut-offs for where most clinical efficacy is considered to be lost [5]. In the absence of specific PhenoSense upper cut-offs for AZT and 3TC, we underlined AZT folds ≥5 fold and 3TC folds ≥100-fold.

### Effects of DRM patterns on predicted NNRTI susceptibility

**Fig 2:**
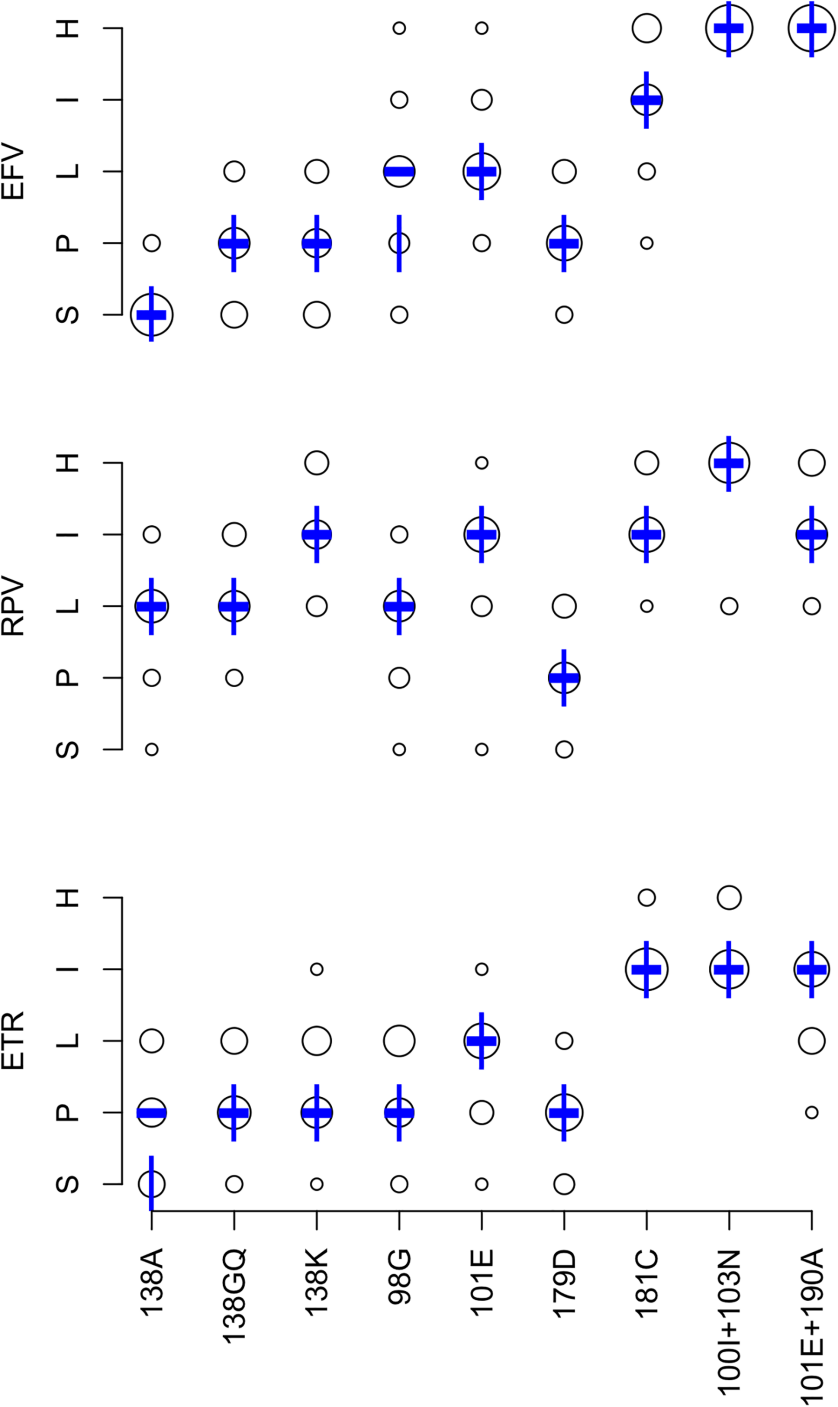
Expert panel assessments of 9 NNRTI-associated drug-resistance mutation (DRM) patterns. Abbreviations: EFV (efavirenz), ETR (etravirine), RPV (riplivirine), S (susceptible), P (potential low-level resistance), L (low-level resistance), I (intermediate resistance), H (high-level resistance). The diameter of each circle is proportional to the number of experts at the assigned level shown on the Y-axis. The bold dash is the median of the expert assessments. The vertical lines represent the HIVDB version 7.0 interpretations.

Fig 2 summarizes the expert panel’s NNRTI interpretations. The completion rate was 91.4% for the 432 DRM pattern-ARV-interpretations (9 patterns x 3 NNRTIs x 16 experts). Originally, 25 (5.8%) pattern-ARV-interpretations differed from the expert median by more than one-level. Following re-evaluation of outliers, 16 (3.7%) pattern-ARV-interpretations differed by more than one level.

The mean absolute deviation from the expert median was 0.43 levels: <0.5 levels for 16 pattern-ARV combinations and 0.5 to 1.0 for 11 pattern-ARV combinations. There was a one-level difference between the expert median and HIVDB 7.0 for two of the 27 pattern-ARV combinations: one for EFV and one for ETR. Table 3 lists these differences and indicates that for each a change was made to the HIVDB scoring such that HIVDB 8.1 yields results that match the expert median.

After updating the scoring system, we determined the relative NNRTI susceptibility profiles of 31,484 viruses in HIVDB with 2,347 distinct NNRTI DRM patterns. Of the 64 possible profiles, 15 occurred in at least 1% of published sequences. Table 6 displays these 15 profiles, which accounted for 98.5% of virus sequences with one or more NNRTI DRMs. The most common profile, high-level EFV resistance and susceptibility to RPV and ETR, occurred in 26.6% of viruses. This profile was caused by K103N ± (P225H or V108I) in 75% of cases. Table 7 summarizes available phenotypic data in HIVDB for the nine NNRTI DRM patterns.

**Table 6:** Patterns of predicted relative NNRTI susceptibility profiles for 31,484 viruses in HIVDB with 2,347 distinct NNRTI-Resistance patterns*.

EFV	RPV	ETR	% of Total Relative NNRTI Susceptibility Profiles^†^	Example DRM Patterns^§^	DRM Pattern % with Profile	DRM Pattern % of Total
High	Susc	Susc	26.6	K103N	61.1	16.2
				K103N,P225H	8.6	2.3
				K103N,V108I	6.4	1.7
High	High	Int	15.2	L100I,K103N	16.9	2.6
				Y181C,G190A	7.9	1.2
				K101E,G190A	6.6	1.0
High	Low	Susc	8.2	K103N,G190A	14.2	1.2
				A98G,K103N	8.9	0.7
				K103S,G190A	8.1	0.7
High	High	High	7.7	K101E,Y181C,G190A	8.4	0.6
				K101P,K103N	7.4	0.6
				K101E,Y181C,G190S	4.1	0.3
Susc	Susc	Susc	7.7	V179D	47.2	3.6
				V179E	22.5	1.7
				V108I	17.9	1.4
Susc	Low	Susc	7.4	E138A	83.6	6.1
				E138G	8.2	0.6
				H221Y	5.9	0.4
Int	Int	Int	4.6	Y181C	75.8	3.5
				V108I,Y181C	11.5	0.5
				Y181C,N348I	2.6	0.1
High	Int	Int	4.4	K103N,Y181C	64.9	2.9
				K103N,V108I,Y181C	5.8	0.3
				G190Q	4.3	0.2
High	High	Susc	3.2	Y188L	72.0	2.3
				K103N,Y188L	15.3	0.5
				V106M,Y188L	4.0	0.1
Int	Low	Susc	2.5	G190A	73.5	1.8
				K103R,V179D	14.4	0.4
				G190A,N348I	2.9	0.1
Int	High	Int	2.5	Y181C,H221Y	39.2	1.0
				V108I,Y181C,H221Y	23.2	0.6
				A98G,Y181C	16.0	0.4
Low	Low	Susc	1.7	A98G	93.4	1.6
				A98G,V108I	2.2	0.0
				V108I,H221Y	2.2	0.0
High	Int	Low	1.7	A98G,G190A	12.8	0.2
				K101E,K103N	5.8	0.1
				K103R,V179D,G190A	5.4	0.1
High	High	Low	1.4	A98G,Y188L	14.8	0.2
				V179D,Y188L	12.4	0.2
				V179E,Y188L	10.8	0.1
Int	Susc	Susc	1.2	K103S	32.5	0.4
				V106A	21.4	0.3
				K238T	20.3	0.2

Footnote

*Obtained from HIVDB (https://hivdb.stanford.edu/dr-summary/pattern-scores/NNRTI/) January 2017. For the purposes of this analysis, the HIVDB interpretations of “Susceptible” and “Potential Low resistance” were grouped together as “Susceptible”.

^**†**^Relative susceptibility patterns accounting for ≥1% of all such patterns are shown. These patterns account for the HIVDB interpretations of 98.5% of sequences containing ≥1 NNRTI DRM.

^**§**^For each relative NRTI susceptibility pattern, the three most common NNRTI DRM patterns responsible for the relative susceptibility pattern are shown as examples.

**Table 7:** In vitro susceptibilities associated with the 9 NNRTI drug resistance mutation patterns.

Overall Pattern*	Specific Pattern*	%Exact^†^	%Included^†^	EFV^§^	ETR^§^	RPV^§^
E138A	E138A	6.15%	8.82%	1.3_14_	2.1_6_	1.8_5_
E138K	E138K	0.30%	0.77%	1.3_3_	2.2_3_	2.3_3_
E138GQ	E138G	0.60%	1.44%	1.3_3_	1.8_2_	1.5_1_
	E138Q	0.09%	1.16%	1.2_3_	-	**2.9**_**1**_
A98G	A98G	1.63%	7.68%	0.7_16_	0.5_4_	-
V179D	V179D	3.62%	6.59%	**3.4**_**5**_	1.2_5_	**2.8**_**1**_
K101E	K101E	0.58%	7.98%	2.1_12_	1.8_7_	1.8_7_
Y181C	Y181C	3.47%	22.08%	1.6_81_	**5.5**_**27**_	2.2_12_
L100I, K103N	L100I, K103N	2.58%	4.26%	**200**_**59**_	**6.8**_**25**_	**14**_**7**_
K101E, G190A	K101E, G190A	1.00%	4.27%	**83**_**9**_	**3.4**_**3**_	**3.1**_**2**_

*Mutation patterns are defined as those matching the listed mutations and not containing additional mutations at positions 100, 101, 103, 106, 181, 188, 190, and 230.

^**†**^Exact: % of sequences exactly matching the mutation pattern; Included: % of sequences matching or including the mutation pattern.

^**§**^Median fold reduced susceptibility as determined by the PhenoSense assay (Monogram Biosciences, South San Francisco). Sequences with electrophoretic mixtures were excluded. “-”indicates that no phenotype results were available for a particular mutation pattern / NNRTI combination. Fold reductions in susceptibility ≥2.5 for RPV and ≥3 for EFV and ETR are in bold. Fold reductions in susceptibility ≥10 are also underlined [5, 11].

### Effects of DRM patterns on predicted PI susceptibility

**Fig 3:**
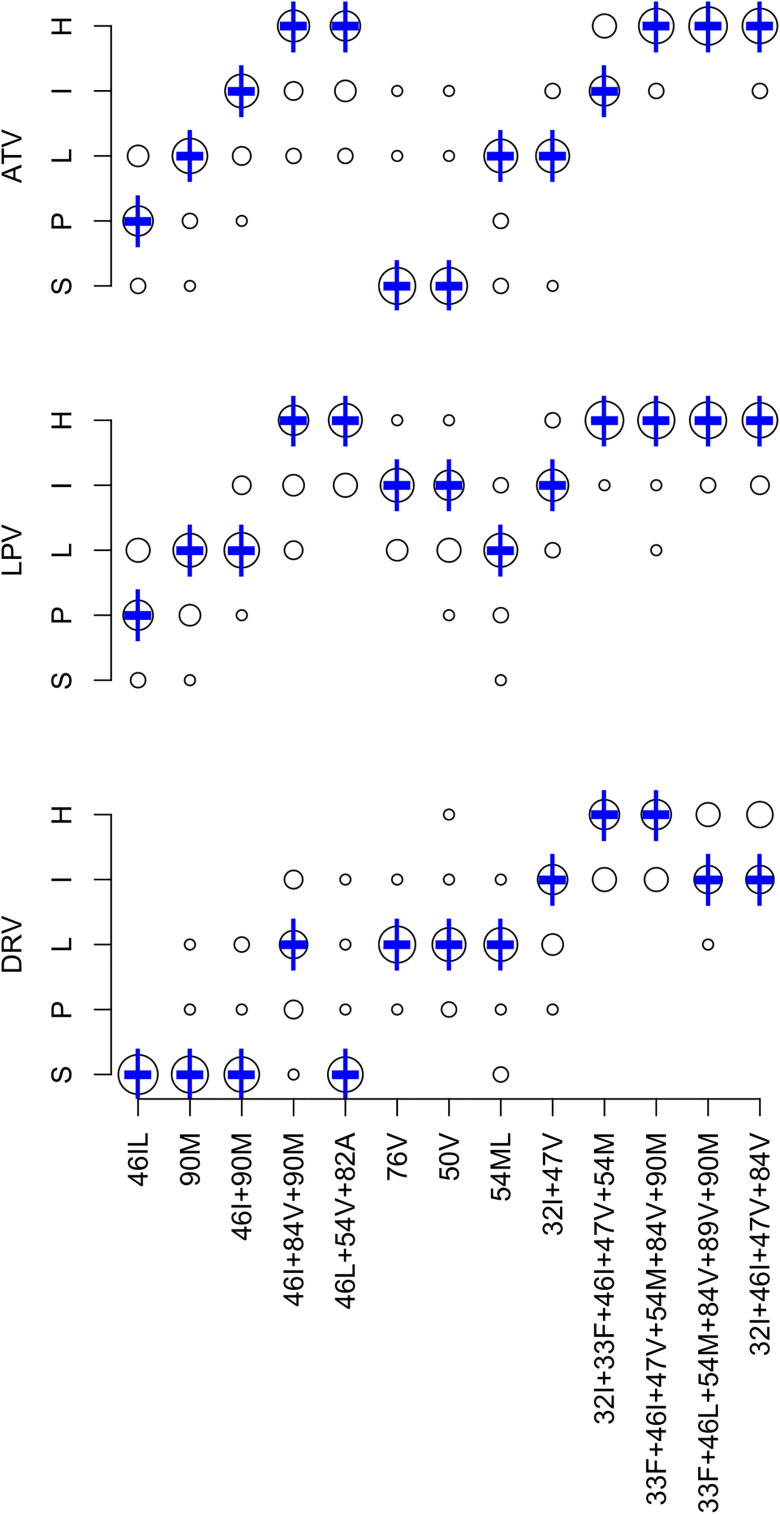
Expert panel assessments of 13 PI-associated drug-resistance mutation (DRM) patterns. Abbreviations: ATV (boosted atazanavir), DRV (boosted darunavir), LPV (boosted lopinavir), S (susceptible), P (potential low-level resistance), L (low-level resistance), I (intermediate resistance), H (high-level resistance). The diameter of each circle is proportional to the number of experts at the assigned level shown on the Y-axis. The bold dash is the median of the expert assessments. The vertical lines represent the HIVDB version 7.0 interpretations.

Fig 3 summarizes the expert panel’s PI interpretations. The completion rate was 87.8% for the 624 DRM pattern-ARV-interpretations (13 patterns x 3 PIs x 16 experts). Originally, 32 (5.1%) pattern-ARV-interpretations differed from the expert median by more than one level. Following re-evaluation of outliers, 19 (3.0%) pattern-ARV-interpretations differed by more than one level. The mean absolute deviation from the median was 0.34 levels: <0.5 levels for 34 pattern-ARV combinations and 0.5 to 1.0 for 5 pattern-ARV combinations. There was no difference between the expert median and HIVDB 7.0 for any of the 39 pattern-ARV combinations.

After updating the scoring system, we determined the relative PI susceptibility profiles of 19,379 virus sequences in HIVDB with 5,634 distinct PI DRM patterns. Of the 64 possible profiles, 14 occurred in at least 1% of the virus sequences. Table 8 displays these 14 patterns, which accounted for 94.6% of sequences. The most common profile was susceptibility to ATV/r, DRV/r, and LPV/r, which was caused by D30N ± N88D, L33F, M46L, or Q58E in nearly 60% of cases. The second most common susceptibility profile was high-level resistance to ATV/r and LPV/r and susceptibility to DRV/r, which was often caused by V82A in combination with M46I/L, I54V, and/or L90M. Table 9 summarizes available phenotypic data for the 13 PI DRM patterns. Incomplete data was available for five of the patterns, most notably L76V alone.

**Table 8:** Patterns of predicted relative PI susceptibility profiles for 19,379 viruses in HIVDB with 5,634 distinct PI-Resistance patterns*.

ATV	LPV	DRV	% of Total Relative PI Susceptibility Profiles^†^	Example DRM Patterns^§^	DRM Pattern % with Profile	DRM Pattern % of Total
Susc	Susc	Susc	18.3	D30N,N88D	17.8	3.3
				L33F	10.4	1.9
				M46L	9.8	1.8
High	High	Susc	17.3	I54V,V82A,L90M	6.0	1.0
				L24I,M46L,I54V,V82A	3.5	0.6
				M46L,I54V,V82A,L90M	2.9	0.5
High	High	Low	16.7	M46I,I84V,L90M	2.6	0.4
				M46I,G73S,I84V,L90M	2.1	0.4
				M46I,G73T,I84V,L90M	1.7	0.3
Low	Low	Susc	8.9	L90M	83.6	7.4
				I54V	3.9	0.4
				D30N,L90M	1.6	0.1
High	High	Int	6.9	I54L,I84V,L90M	1.0	0.1
				L33F,I54L,G73T,I84V,L90M	0.9	0.1
				M46I,L76V,I84V	0.7	0.1
Int	Low	Susc	6.0	M46I,L90M	23.1	1.4
				G73S,L90M	17.6	1.1
				K20T,L90M	13.6	0.8
High	Int	Low	4.0	I84V,L90M	15.5	0.6
				G73S,I84V,L90M	13.4	0.5
				I84V	6.8	0.3
High	High	High	3.8	V32I,K43T,M46I,I47V,I54M,V82A,L90M	1.3	0.1
				V32I,L33F,M46I,I47V,I54M,V82A,L90M	0.9	0.0
				L10F,V11I,K20T,V32I,L33F,I54V,G73S,I84V,L89V,L90M	0.8	0.0
Int	Int	Susc	3.7	I54V,V82A	30.0	1.1
				V82A,L90M	7.7	0.3
				M46L,V82A	7.3	0.3
High	Int	Susc	3.2	M46I,G73S,L90M	16.7	0.5
				M46I,G73T,L90M	6.3	0.2
				M46I,F53L,G73S,L90M	3.4	0.1
Low	Susc	Susc	1.8	K20T,D30N,N88D	25.5	0.5
				D30N,L33F,N88D	15.6	0.3
				D30N,M46I,N88D	13.0	0.2
High	Susc	Susc	1.5	N88S	27.9	0.4
				M46I,N88S	19.5	0.3
				I50L	8.4	0.1
Low	Int	Susc	1.4	V82A	80.5	1.1
				L10F,V82A	4.1	0.1
				L24I,V82A	3.0	0.0
Int	High	Susc	1.1	L24I,I54V,V82A	22.3	0.2
				L24I,M46L,V82A	14.6	0.2
				L10F,I54V,V82A	12.6	0.1

*Obtained from HIVDB (https://hivdb.stanford.edu/dr-summary/pattern-scores/PI/) January 2017. For the purposes of this analysis, the HIVDB interpretations of “Susceptible” and “Potential Low resistance” were grouped together as “Susceptible”.

^**†**^Relative susceptibility patterns accounting for ≥1% of all such patterns are shown. These patterns account for the HIVDB interpretations of 94.6% of sequences containing ≥1 PI DRM.

^**§**^For each relative PI susceptibility pattern, the three most common PI DRM patterns responsible for the relative susceptibility pattern are shown as examples.

**Table 9:** In vitro susceptibilities associated with the 13 PI drug resistance mutation patterns.

Overall Pattern*	Specific Pattern*	%Exact^†^	%Included^†^	LPV^§^	ATV^§^	DRV^§^
N46IL	M46I	1.66%	32.22%	2.7_3_	2.2_3_	0.8_2_
	M46L	1.79%	14.67%	1.6_2_	2.4_1_	0.7_1_
L90M	L90M	7.43%	50.86%	1.6_42_	**3.1**_**33**_	0.9_19_
M46I, L90M	M46I, L90M	1.38%	19.36%	2.7_23_	**4.9**_**17**_	0.8_6_
M46I, I84V, L90M	M46I, I84V, L90M	0.43%	7.65%	**17**_**27**_	**22**_**19**_	4_4_
M46L, I54V, V82A	M46L, I54V, V82A	0.41%	5.99%	**37**_**17**_	**31**_**9**_	1.7_4_
L76V^¶^	L76V^¶^	0.09%	4.15%	-	-	-
I50V	I50V	0.12%	2.63%	6.7_4_	1.4_2_	-
I54LM	I54L	0.15%	4.50%	4.6_1_	1.7_1_	3.6_1_
	I54M	0.07%	3.74%	2.9_1_	**3.4**_**1**_	-
V32I, I47V	V32I, I47V	0.08%	4.17%	4_1_	**6.3**_**1**_	1.3_1_
V32I, M46I, I47V, I84V	V32I, M46I, I47V, I84V	0.01%	0.75%	**77**_**2**_	**40**_**2**_	**25**_**1**_
L33F, M46I, I47V, I54M, 84V, 90M	L33F, M46I, I47V, I54M, 84V, 90M	0.01%	0.02%	**175**_**6**_	**74**_**6**_	**>200**_**3**_
V32I, L33F, M46I, I47V, I54M	V32I, L33F, M46I, I47V, I54M	0.01%	0.61%	-	-	-
L33F, M46L, I54M, I84V, V89I, L90M	L33F, M46L, I54M, I84V, V89I, L90M	0.00%	0.00%	**43**_**1**_	-	-

*Mutation patterns are defined as those matching the listed mutations and not containing additional mutations at positions 30, 32, 46, 47, 48, 50, 54, 76, 82, 84, 88, and 90.

^**†**^Exact: % of sequences exactly matching the mutation pattern; Included: % of sequences matching or including the mutation pattern.

^**§**^Fold reduced susceptibility as determined by the PhenoSense assay (Monogram Biosciences, South San Francisco). Sequences with electrophoretic mixtures were excluded. “-”indicates that no phenotype results were available for a particular mutation pattern / PI combination. Fold reductions in susceptibility ≥3 for ATV, ≥9 for LPV, and ≥10 for DRV are in bold [5, 8, 9]. Fold reductions in susceptibility ≥6 for ATV, ≥40 for LPV, and ≥90 for DRV are also underlined [5, 8, 9].

^¶^Of 67 LPV susceptibility results in HIVDB on viruses containing L76V, all had one or more additional DRMs.

### Effects of DRM patterns on predicted INSTI susceptibility

**Fig 4:**
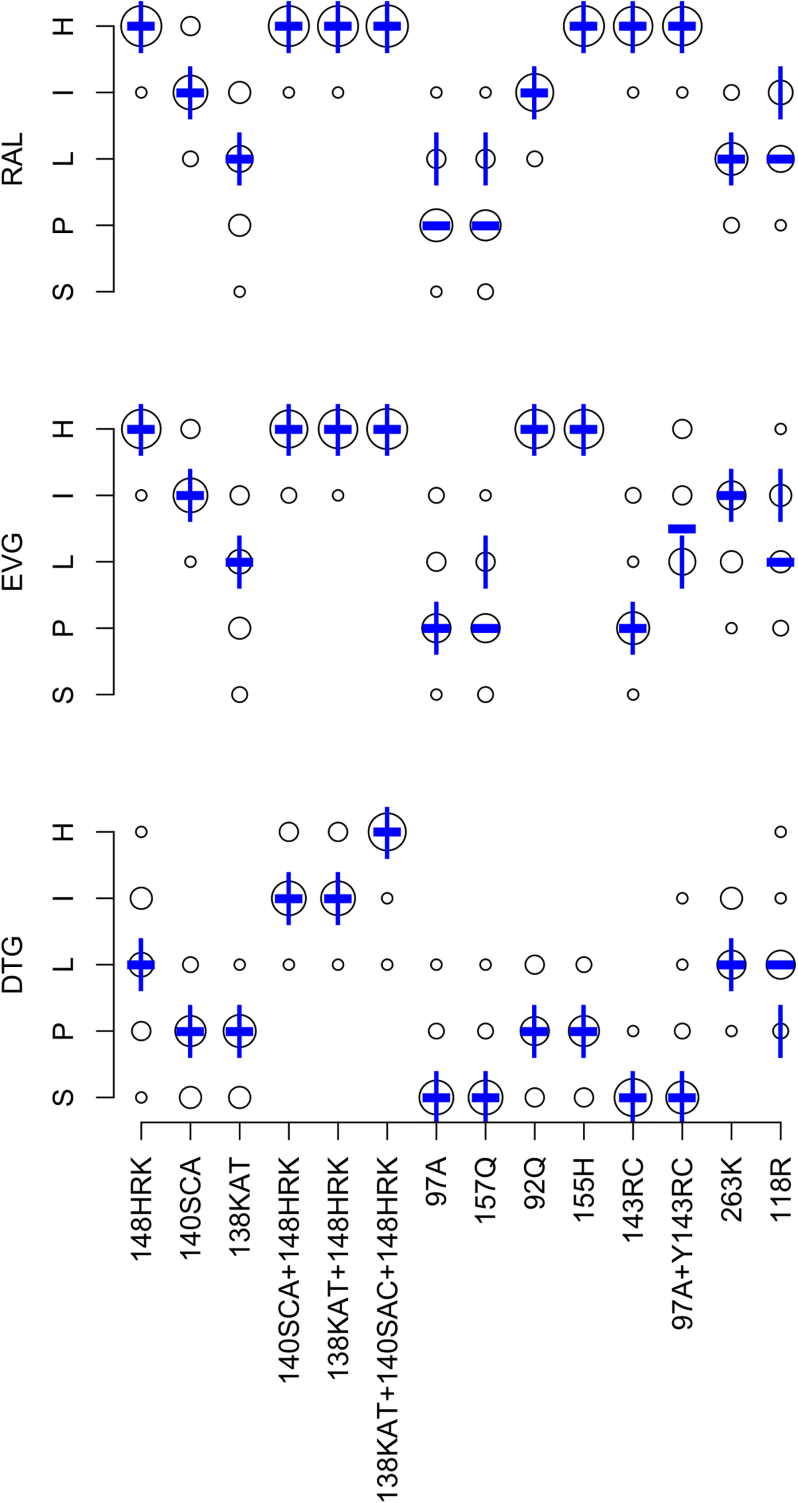
Expert panel assessments of 14 INSTI-associated drug-resistance mutation (DRM) patterns. Abbreviations: DTG (dolutegravir), EVG (elvitegravir), raltegravir (RAL), S (susceptible), P (potential low-level resistance), L (low-level resistance), I (intermediate resistance), H (high-level resistance). The diameter of each circle is proportional to the number of experts at the assigned level shown on the Y-axis. The bold dash is the median of the expert assessments. The vertical lines represent the HIVDB version 7.0 interpretations.

Fig 4 summarizes the expert panel’s INSTI interpretations. The completion rate was 85.0% for the 672 DRM pattern-ARV-interpretations (14 patterns x 3 INSTIs x 16 experts). Originally, 22 (3.2%) pattern-ARV-interpretations differed from the expert median by more than one level. Following re-evaluation of outliers, 16 (2.3%) pattern-ARV-interpretations differed by more than one level.

The mean absolute deviation from the median was 0.31 levels: <0.5 levels for 33 pattern-ARV combinations and 0.5 to 1.0 for nine pattern-ARV combinations. There was a one-level difference between the expert median and HIVDB 7.0 for six of the 42 pattern-ARV combinations: three for RAL, two for EVG, and one for DTG. Table 3 lists these differences and indicates that for four of these a change was made in the HIVDB scoring such that HIVDB 8.1 system yields results matching the expert median.

After updating the scoring system, we determined the relative INSTI susceptibility profiles of 1,536 viruses in HIVDB with 209 distinct INSTI DRM patterns. Of the 64 possible profiles, 11 occurred in at least 1% of the virus sequences. Table 10 displays these 11 profiles, which accounted for 97.7% of virus sequences. The most common susceptibility profile was susceptibility to all INSTIs, which was caused by either E157Q, T97A, or Q95K alone. The second most common INSTI profile–high-level RAL and EVG resistance with intermediate (18.4%) or low-level (17.1%) DTG resistance–was caused by G140S + Q148H in 67% of cases. Table 11 summarizes available phenotypic data for the 14 INSTI DRM patterns. For many patterns, few data were available for DTG.

**Table 10:** Patterns of relative INSTI susceptibility for 1,536 viruses in HIVDB with 209 distinct INSTI-Resistance patterns*.

RAL	EVG	DTG	% of Total Relative INSTI Susceptibility Profiles^†^	Example DRM Patterns^§^	DRM Pattern % with Profile		DRM Pattern % of Total
Susc	Susc	Susc	34.9	E157Q	65.7		22.9
				T97A	32.3		11.3
				Q95K	2.1		0.7
High	High	Int	18.4	G140S,Q148H	66.7		12.3
				G140S,Q148R	3.5		0.6
				E138K,Q148R	3.2		0.6
High	High	Susc	17.1	N155H	55.5		9.5
				N155H,G163R	12.5		2.1
				N155H,E157Q	12.2		2.1
Low	Low	Susc	6.5	G163R	30		2.0
				E138K	21		1.4
				G163K	17		1.1
High	High	High	5.3	E138A,G140S,Q148H	23.2		1.2
				E138K,G140S,Q148H	18.3		1.0
				E138T,G140S,Q148H	6.1		0.3
High	High	Low	4.5	E92Q,N155H	23.2		1.0
				Q148R	14.5		0.7
				Y143C,N155H,S230R	7.2		0.3
High	Int	Susc	3.4	L74M,T97A,Y143R	15.4		0.5
				T97A,Y143C,S230R	11.5		0.4
				T97A,Y143R,G163R	11.5		0.4
High	Low	Susc	2.2	T97A,Y143R	58.8		1.3
				L74M,Y143R	20.6		0.5
				T97A,Y143C	8.8		0.2
Int	High	Susc	1.8	E92Q	55.6		1.0
				E92Q,T97A	14.8		0.3
				T66A,G163R	7.4		0.1
High	Susc	Susc	1.4	Y143R	71.4		1.0
				Y143C	9.5		0.1
				Y143S	9.5		0.1
Low	Int	Low	1.2	R263K	94.4		1.1
				E157Q,R263K	5.6		0.1
High	Int	Low	1	L74M,T97A,Y143C,S230R	25		0.3
				L74I,T97A,Y143C,S230R	12.5		0.1
				Y143C,G163R,S230R	12.5		0.1

*Obtained from HIVDB (https://hivdb.stanford.edu/dr-summary/pattern-scores/INSTI/) January 2017. For the purposes of this analysis, the HIVDB interpretations of “Susc” and “Potential Low resistance” were grouped together as “Susc”.

^**†**^Relative susceptibility patterns accounting for ≥1% of all such patterns are shown. These patterns account for the HIVDB interpretations of 97.7% of sequences containing ≥1 INSTI DRM.

^**§**^For each relative INSTI susceptibility pattern, the three most common INSTI DRM patterns responsible for the relative susceptibility pattern are shown as examples.

**Table 11:** In vitro susceptibilities associated with the 14 INSTI drug resistance mutation patterns.

Overall Pattern*	Specific Patterns*	%Exact^†^	%Include^†^	RAL^§^	EVG^§^	DTG^§^
Q148HRK	Q148H	0.20%	17.58%	**19**_**8**_	**5.3**_**3**_	0.5_3_
	Q148R	0.65%	5.99%	**30**_**10**_	**109**_**5**_	1.1_3_
	Q148K	0.00%	0.85%	**40**_**6**_	**67**_**3**_	1.5_3_
G140SCA	G140S	0.07%	19.99%	1.6_3_	**4.5**_**2**_	0.8_1_
	G140C	0.00%	0.39%	1.1_1_	**6.1**_**1**_	0.5_1_
	G140A	0.00%	1.37%	2.7_1_	**5.4**_**1**_	0.7_1_
E138KAT	E138K	1.37%	5.53%	0.9_3_	0.7_3_	0.9_1_
	E138A	0.20%	2.15%	1.1_1_	1.3_1_	0.9_1_
G140SCA, Q148HRK	G140S, Q148H	12.24%	17.32%	**>150**_**18**_	**>150**_**9**_	**3.6**_**7**_
	G140S, Q148R	0.65%	1.82%	**>150**_**10**_	**>150**_**3**_	**8.4**_**7**_
	G140S, Q148K	0.00%	0.52%	**4.4**_**4**_	**117**_**2**_	1.5_1_
	G140A, Q148H	0.00%	0.00%	**>150**_**1**_	-	-
	G140A, Q148R	0.39%	1.04%	**96**_**5**_	**100**_**3**_	**13**_**1**_
	G140A, Q148K	0.07%	0.33%	**>150**_**1**_	-	-
	G140C, Q148R	0.00%	0.39%	**114**_**2**_	**>150**_**2**_	**4.9**_**1**_
E138KAT, Q148HRK	E138A, Q148R	0.00%	0.33%	**110**_**1**_	**>150**_**1**_	2.6_1_
	E138K, Q148H	0.00%	0.98%	**19**_**2**_	**6.7**_**1**_	0.9_1_
	E138K, Q148R	0.59%	2.15%	**75**_**7**_	**>150**_**3**_	**3.5**_**4**_
	E138K, Q148K	0.00%	0.26%	**>150**_**5**_	**>150**_**2**_	**13**_**3**_
E138KAT, G140SAC, Q148HRK	E138K, G140A, Q148R	0.00%	0.13%	**>150**_**2**_	**>150**_**2**_	-
	E138K, G140S, Q148H	0.98%	0.98%	**>150**_**3**_	**>150**_**1**_	**8.4**_**3**_
	E138K, G140S, Q148R	0.07%	0.20%	**>150**_**1**_	-	**8.3**_**1**_
T97A	T97A	11.26%	21.16%	1.3_13_	**5.4**_**13**_	0.9_5_
E157Q	E157Q	22.92%	28.39%	1.1_17_	1.8_18_	1.1_2_
E92Q	E92Q	0.98%	3.71%	**4.6**_**20**_	**33**_**14**_	1.5_3_
N155H	N155H	9.51%	21.61%	**17**_**23**_	**45**_**17**_	1.8_8_
Y143RC	Y143R	0.98%	5.53%	**20**_**7**_	2.9_4_	1.3_1_
	Y143C	0.13%	2.99%	**4.4**_**7**_	1.9_6_	0.8_1_
T97A, Y143RC	T97A, Y143R	1.30%	3.52%	**>150**_**12**_	**32**_**6**_	1_3_
	T97A, Y143C	0.20%	1.50%	**76**_**9**_	**4.9**_**7**_	1_4_
R263K	R263K	1.11%	1.30%	1.1_3_	**4.6**_**3**_	1.9_2_
G118R	G118R	0.00%	0.00%	-	-	-

*Mutation patterns are defined as those matching the listed mutations and not containing additional mutations at positions 66, 92, 97, 118, 121, 138, 140, 143, 147, 148, 155, and 263.

^**†**^Exact: % of sequences exactly matching the mutation pattern; Included: % of sequences matching or including the mutation pattern.

^**§**^Fold reduced susceptibility as determined by the PhenoSense assay (Monogram Biosciences, South San Francisco). Sequences with electrophoretic mixtures were excluded. “-”indicates that no phenotype results were available for a particular mutation pattern / INSTI combination. Fold reductions in susceptibility ≥3 fold are in bold [12, 13]. Fold reductions in susceptibility ≥10-fold are also underlined [12, 13].

## Discussion

Decision support systems have become increasingly important for the interpretation of genetic sequences for clinical purposes. Such systems comprise rule-based systems designed to emulate consultation with a subject-matter expert and machine-learning systems that use an algorithm to arrive at an optimized result through the analysis of a large dataset. Machine-learning systems are useful for scenarios in which sufficient amounts of appropriate raw data are available for algorithm training and validation. Rule-based systems are useful for scenarios that require knowledge for which the raw data are either not available or are too heterogeneous to combine in a manner amenable to machine learning. Compared with machine-learning systems, rule-based systems have the advantage of being transparent and educational but the disadvantage of being subjective.

There are several machine-learning systems for HIV-1 GRT interpretation that use proprietary datasets containing either large numbers of correlations between viral genotype and phenotype [16–20] or between genotype and the virological response to a new treatment regimen [21–25]. However, rule-based systems have been used more commonly than machine-learning systems for HIV-1 GRT interpretation due to their transparency, ability to take into account diverse forms of data, and ability to represent expert opinion [26–31].

Rule-based systems represent knowledge in the form of IF/THEN rules in which the IF clause specifies a condition to be evaluated and the THEN clause specifies an action to be taken. In the HIVDB GRT-IS, one type of condition–the presence of a particular DRM–triggers a comment about that DRM. A second type of condition–the sum of DRM penalty scores associated with an ARV–triggers the assignment of a predicted level of susceptibility for that ARV. In this paper, we described the HIVDB GRT-IS and summarized the authors’ opinions on several aspects of HIV-1 GRT interpretation, including which ARVs should be included in an analysis and how various combinations of DRMs interact to influence ARV susceptibility.

### ARVs and pharmacologic considerations

There was universal agreement within the expert panel that a GRT report should include the following five NRTs (3TC, ABC, AZT, FTC, and TDF), four NNRTIs (EFV, ETR, NVP, and RPV), three PIs with pharmacologic boosting (ATV/r, ATV/c, DRV/r, DRV/c, and LPV/r), and three INSTIs (DTG, EVG, and RAL). There was a range of opinion on whether the NRTIs d4T and ddI and the PIs NFV, FPV/r, IDV/r, SQV/r, and TPV/r should continue to be included because the use of these ARVs is no longer recommended in all but a few clinical situations. In response to this feedback, HIVDB users are now provided the option of excluding ddI, d4T, IDV/r, SQV/r, FPV/r, and TPV/r.

The panel agreed that TDF and TAF should receive similar mutation penalty scores but stipulated that this decision should be re-evaluated if the greater intracellular levels of tenofovir produced by TAF could be shown to be clinically significant in the presence of reduced *in vitro* tenofovir susceptibility [32, 33]. In response to the panel’s recommendation to indicate which of the two DRV/r and DTG dosing schedules should be used, the HIVDB output was modified to include comments indicating that the higher dosing schedule should be used in the presence of low-level, intermediate, or high-level resistance to these ARVs [12, 14, 34].

### DRM patterns: Overall concordance

There was a high level of concordance in the interpretation of 48 DRM test patterns. Only 5.5%, 5.8%, 5.1%, and 3.2% of NRTI, NNRTI, PI, and INSTI interpretations, respectively, were considered outliers. These proportions were reduced to 3.8%, 3.7%, 4.8%, and 2.3%, respectively after the outliers were re-reviewed by panel members. The proportion of outliers, however, would likely have been higher if panel members did not have the opportunity to ignore patterns of which they were uncertain (3% to 15% depending on the ARV class).

The median expert level differed from HIVDB 7.0 by one level for 12 (23.1%) of the 52 NRTI DRM pattern-ARV interpretations, two of the 27 NNRTI DRM pattern-ARV interpretations, none of the 39 PI DRM pattern-ARV interpretations, and six (14.2%) of the 42 INSTI DRM pattern-ARV interpretations. For 18 of the 20 differences, the HIVDB GRT scoring was modified so that the interpretation matched the expert median. The following paragraphs summarize the considerations leading to the changes to the HIVDB scoring system as they pertain to the 48 DRM test patterns.

### NRTI DRM patterns

In HIVDB 7.0, K65R+M184V was assigned high-level TDF resistance because of the frequency of this DRM pattern in individuals with VF on a TDF-containing regimen [35]. However, phenotypic data indicate that viruses with this DRM pattern have a median reduction in susceptibility of just 1.2 fold (Table 5), which is below the 1.4 fold PhenoSense cut-off for the start of low-level TDF [4, 7]. The minimal reduction in TDF susceptibility caused by K65R+M184V, reflects the approximately two-fold reduction in susceptibility caused by K65R counteracted in part by the nearly two-fold increase in susceptibility caused by M184V [36, 37]. Awareness of these data by the expert panel led to the updated interpretation of intermediate TDF resistance for K65R + N184V for HIVDB 8.1. Despite this change, AZT remains the NRTI of choice for viruses with K65R+M184V because this DRM pattern increases AZT susceptibility (Table 5).

In HIVDB 7.0, the combination of the three type 1 TAMs—M41L, L210W, and T215Y —led to the assignment of high-level TDF resistance because this pattern is associated with about 4.0 fold reduced TDF susceptibility (Table 5) [36] and with a minimal (approximately 0.2 log) reduction in VL following TDF intensification [38]. Expert panel polling led to the revised assignment of intermediate TDF resistance for this DRM pattern to indicate that TDF, particularly when used in combination with 3TC or FTC, may retain ARV activity against viruses with this DRM pattern and may be useful for salvage therapy. Of note, a study published during the preparation of this manuscript reported that the presence of the two Type 1 TAMs, M41L and L210W (without T215YF) did not appear to influence the response to a first-line TDF-or TAF-containing regimen in nine clinical trials conducted by Gilead Sciences from 2000 to 2013 [39]. This study did not examine the response to therapy of viruses containing all three type 1 TAMs, because individuals with these viruses were excluded from trial enrollment.

The HIVDB GRT-IS predicts that the NRTIs TDF, 3FTC, and AZT often have different susceptibility profiles because K65R and the 3FTC-resistance DRM M184V are associated with increased ARV susceptibility (the former to AZT and the latter to AZT and TDF). Although ABC has similar activity as TDF *in vivo* [40, 41], it is infrequently predicted to more active because each of the TDF-associated DRMs confer ABC cross-resistance and because M184V and L74V are associated with reduced susceptibility to ABC but not TDF. The NRTI-backbone of ABC-3TC has also been associated with a higher risk of VF than TDF-FTC in patients with plasma HIV-1 RNA levels exceeding 100,000 copies/ml [42].

### NNRTI DRM patterns

Few changes were made to the NNRTI scoring system (Table 3). The cross-resistance profiles shown in Table 6 indicate that viruses with high-level resistance to ETR will usually be cross-resistant to EFV and RPV. Although RPV has a similar chemical structure to ETR, its genetic barrier to clinically significant resistance is lower than ETR because it is administered at one-sixteenth of ETR’s dose [43].

RPV was generally, but not always, predicted to be more active than EFV. Specifically, EFV was predicted to be more active than RPV against viruses with E138A/G/Q/K mutations, which are among the most common DRMs in individuals with VF on an RPV-containing regimen [43] and which are associated with greater reductions in susceptibility to RPV than EFV (Table 7).

Although Y181C alone retains greater *in vitro* susceptibility to EFV compared with RPV and ETR (Table 7), it yielded interpretations of intermediate resistance to each of the NNRTIs (Table 6) because past studies have shown that EFV had reduced efficacy at treating viruses from patients with past NNRTI experience even if genotypic resistance testing demonstrated Y181C alone [44–46].

The polymorphic mutation V179D appears to cause low-level reductions in EFV and RPV susceptibility. However, one retrospective study reported that V179D does not increase the risk of virological failure on a first-line EFV-containing regimen [47].

### PI DRM patterns

There were no differences between HIVDB 7.0 and the expert panel median for the 13 PI DRM patterns. This high level of agreement may be partly due to findings from several well-powered studies that led to the development of genotypic susceptibility scores for the prediction of *in vitro* susceptibility and *in vivo* response to salvage therapy with LPV/r and DRV/r-containing regimens [48–51]. Fewer data, however, are available for the genotypic predictors of *in vitro* ATV susceptibility and the virological response to ATV/r-containing regimens [52, 53].

The HIVDB GRT-IS PI susceptibility profiles predict that most viruses with high-level DRV resistance will also have high-level resistance to the remaining PIs. Although LPV/r, like DRV/r, has a high genetic barrier to resistance, no viruses were predicted to be more resistant to DRV/r than to LPV/r because each of the DRV-associated DRMs confers cross-resistance to LPV. When used in PI-naïve individuals, ATV and ATV/r usually select for DRMs rarely seen with other PIs, namely I50L and N88S. However, the phenotypic data in Table 9 indicate that many other DRM patterns cause clinically significant reductions in ATV/r susceptibility. Indeed, this may not have been fully appreciated by the expert panel. For example, the DRM pattern V32I+I47V was evaluated as having low-level, intermediate, and intermediate resistance to ATV/r, DRV/r, and LPV/r, respectively. However, the phenotypic data in Table 9 indicated 6.3-fold, 1.3-fold, and 4.0-fold reduced susceptibility to ATV, DRV, and LPV, respectively.

### INSTI DRM patterns

T97A and E157Q are polymorphic accessory INSTI-selected DRMs that occur in 1 to 5% of viruses from untreated persons depending on subtype [54–58]. T97A had previously been reported to be synergistic with Y143C at reducing RAL susceptibility [59]. Based on expert polling, the individual DRM penalty scores for RAL and EVG for T97A and E157Q were each lowered from low-level to potential low-level resistance. In our *post-hoc* analysis of phenotypic data in HIVDB, E157Q alone was not found to reduce RAL and EVG susceptibility, whereas T97A alone was found to reduce EVG susceptibility by about five fold (Table 11). However, in a study published during the completion of this manuscript, the presence of T97A at baseline was reported to not interfere with virological response to therapy with a first-line EVG-containing regimen [60].

R263K is selected *in vitro* by DTG and is associated with about two-fold reduction in susceptibility to DTG [61, 62] (Table 11). R263K has also been reported in previously INSTI-naïve but ARV-experienced patients receiving DTG [63, 64] and in patients receiving DTG monotherapy [65]. Considering the rarity of R263K in patients receiving DTG, its minimal reduction in DTG susceptibility, and its association with reduced replication fitness [66], the expert panel decided to leave the assignment of low-level DTG resistance unchanged from HIVDB 7.0.

G118R is even more rare than R263K. It has been reported in two patients receiving DTG monotherapy [67]. Depending on the study, site-directed mutants with G118R have been reported to be fully susceptible or to have a more than five-fold reduction in susceptibility to each of the INSTIs [67–71]. However, there are no publicly available phenotypic data performed using the PhenoSense assay for isolates with G118R. Given the rarity and uncertainty surrounding the clinical significance of this DRM, the RAL and EVG assignments were left unchanged at intermediate resistance and the DTG assignment was increased from potential low-level to low-level resistance.

Q148H/R/K are nonpolymorphic DRMs selected in patients receiving RAL and EVG that alone reduce RAL and EVG susceptibility by five- to 20-fold. These DRMs usually occur in combination with G140S/A/C or E138K/A/T, in which case they confer a more than 100-fold reduction in susceptibility to RAL and EVG. Q148H/R/K alone are not usually associated with reduced DTG susceptibility. However, in combination with G140S/A/C or E138K/A/T, susceptibility may be reduced by two- to 10-fold [6, 72, 73]. The presence of DRMs at all three positions is associated with a greater than 10-fold reduction in DTG susceptibility. Among individuals receiving DTG In the VIKING-3 study, virological suppression was attained in 10 of 18 with Q148H/R + G140A/S without additional INSTI DRMs but in only three of 17 with Q148H/R in combination with two or more INSTI DRMs (most commonly G140 + E138 mutations) [12, 56]. Considering the importance of DTG in combatting the HIV pandemic, it is essential for there to be more published *in vitro* DTG susceptibility data.

### Limitations and future directions

Many studies describe how DRMs influence ARV efficacy such as whether they are selected by the ARV, whether they influence the *in vitro* activity of an ARV, and whether they influence the virological response to a regimen containing the ARV. Darwinian logic suggests that if a DRM is selected by an ARV, it likely reduces susceptibility to that ARV. Moreover, if individuals with VF while receiving an ARV regimen frequently develop the same DRM in the absence of other DRMs, then the evidence linking the DRM to ARV resistance is stregnthened.

*In vitro* susceptibility data is the main quantitative form of HIV-1 drug resistance data. However, genotype-phenotype correlations cannot always directly used to guide therapy. For example, the RT mutation M184V causes high-level resistance to 3TC and FTC, but most guidelines do not recommend discontinuing these ARVs when choosing a new ARV regimen in patients with this DRM [34, 74]. Conversely, although some DRMs have a minimal effect on ARV susceptibility when they occur alone, these DRMs can be markers for the presence of other DRMs likely to emerge with continued selective drug pressure.

Correlations between genotype and virological outcome have been obtained in clinical trials and retrospective cohort studies. These studies have usually been complicated by the many variables that influence the virological response to a change in ART such as the previous ARVs received, the baseline virus load and CD4 count, the ARVs in the new treatment regimen, and adherence to therapy. Moreover, many of these studies have been confounded by the fact that baseline GRT results were used to guide therapy. Nonetheless, several of these studies produced highly useful data including the influence of many NRTI-associated DRMs on the virological response to regimens containing ABC and TDF [38, 75], the influence of PI DRMs on response to regimens containing LPV/r and DRV/r [8, 48, 50], the influence of NNRTI-associated DRMs on the response to therapy with the etravirine [76], and the influence of INSTI DRMs on the response to therapy with DTG [56].

However, despite the large amount of published drug resistance data, it is often not possible to validate the interpretations for uncommon DRM patterns To handle such scenarios, the HIVDB system contains penalties for many individual DRMs and for several of the most commonly occurring DRM combinations. By adding the individual and combination DRM penalties, the HIVDB system develops interpretations for complex DRM patterns that are based on the more reliable data associated with simpler DRM patterns. The HIVDB GRT-IS is subjective in that It relies on expert opinion to prioritize the relative importance of the various forms of HIV drug resistance data summarized in the preceding paragraphs. The transparency of the HIVDB GRT IS renders it amenable to user feedback and to the type of expert review described in this manuscript. The review led to a series of changes to the HIVDB GRT report and IS and identified many areas of consensus and several areas requiring additional research.

## Supporting information

S1 File The expert panel questionnaire. (DOCX)

## References

[1] Vandamme AM, Camacho RJ, Ceccherini-Silberstein F, de Luca A, Palmisano L, Paraskevis D, et al European recommendations for the clinical use of HIV drug resistance testing: 2011 update. AIDS reviews. 2011;13(2):77–108. . 21587341

[2] Liu TF, Shafer RW. Web resources for HIV type 1 genotypic-resistance test interpretation. Clinical infectious diseases: an official publication of the Infectious Diseases Society of America. 2006;42(11):1608–18. doi: 10.1086/503914 ; PubMed Central PMCID: PMCPMC2547473. 16652319 10.1086/503914 PMC2547473

[3] Tang MW, Liu TF, Shafer RW. The HIVdb system for HIV-1 genotypic resistance interpretation. Intervirology. 2012;55(2):98–101. doi: 10.1159/000331998 . 22286876 10.1159/000331998 PMC7068798

[4] Petropoulos CJ, Parkin NT, Limoli KL, Lie YS, Wrin T, Huang W, et al A novel phenotypic drug susceptibility assay for human immunodeficiency virus type 1. Antimicrobial agents and chemotherapy. 2000;44(4):920–8. Epub 2000/03/18. ; PubMed Central PMCID: PMC89793. 10722492 10.1128/aac.44.4.920-928.2000 PMC89793

[5] Monogram Biosciences. PhenoSense HIV Drug Resistance Assay Report Template (last accessed May 18, 2017). https://wwwmonogrambiocom/sites/monogrambio/files/imce/uploads/PS_report_new_Watermarkpdf. 2017.

[6] Kobayashi M, Yoshinaga T, Seki T, Wakasa-Morimoto C, Brown KW, Ferris R, et al In Vitro antiretroviral properties of S/GSK1349572, a next-generation HIV integrase inhibitor. Antimicrobial agents and chemotherapy. 2011;55(2):813–21. Epub 2010/12/01. doi: 10.1128/AAC.01209-10 ; PubMed Central PMCID: PMC3028777. 21115794 10.1128/AAC.01209-10 PMC3028777

[7] Parkin NT, Hellmann NS, Whitcomb JM, Kiss L, Chappey C, Petropoulos CJ. Natural variation of drug susceptibility in wild-type human immunodeficiency virus type 1. Antimicrobial agents and chemotherapy. 2004;48(2):437–43. Epub 2004/01/27. PubMed Central PMCID: PMC321508. doi: 10.1128/AAC.48.2.437-443.2004 14742192 10.1128/AAC.48.2.437-443.2004 PMC321508

[8] Kempf DJ, Isaacson JD, King MS, Brun SC, Sylte J, Richards B, et al Analysis of the virological response with respect to baseline viral phenotype and genotype in protease inhibitor-experienced HIV-1-infected patients receiving lopinavir/ritonavir therapy. Antivir Ther. 2002;7(3):165–74. Epub 2002/12/19. . 12487383

[9] Naeger LK, Struble KA. Effect of baseline protease genotype and phenotype on HIV response to atazanavir/ritonavir in treatment-experienced patients. AIDS. 2006;20(6):847–53. doi: 10.1097/01.aids.0000218548.77457.76 . 16549968 10.1097/01.aids.0000218548.77457.76

[10] Winters B, Montaner J, Harrigan PR, Gazzard B, Pozniak A, Miller MD, et al Determination of clinically relevant cutoffs for HIV-1 phenotypic resistance estimates through a combined analysis of clinical trial and cohort data. J Acquir Immune Defic Syndr. 2008;48(1):26–34. doi: 10.1097/QAI.0b013e31816d9bf4 . 18360290 10.1097/QAI.0b013e31816d9bf4

[11] Picchio G, Vingerhoets J, Tambuyzer L, Coakley E, Haddad M, Witek J. Short communication prevalence of susceptibility to etravirine by genotype and phenotype in samples received for routine HIV type 1 resistance testing in the United States. AIDS research and human retroviruses. 2011;27(12):1271–5. doi: 10.1089/aid.2011.0049 . 21557669 10.1089/AID.2011.0049

[12] FDA. Tivicay (dolutegravir) prescribing information. http://wwwaccessdatafdagov/drugsatfda_docs/label/2013/204790lblpdf. 2015.

[13] Monogram Biosciences. PhenoSense Integrase template report. https://wwwmonogrambiocom/sites/monogrambio/files/imce/uploads/PSINT%2BDTGpdf. 2017.

[14] FDA. Prezista (Darunavir) Prescribing Information. https://wwwprezistacom/sites/default/files/pdf/us_package_insertpdf. 2016.

[15] Rhee SY, Varghese V, Holmes SP, Van Zyl GU, Steegen K, Boyd MA, et al Mutational Correlates of Virological Failure in Individuals Receiving a WHO-Recommended Tenofovir-Containing First-Line Regimen: An International Collaboration. EBioMedicine. 2017;18:225–35. doi: 10.1016/j.ebiom.2017.03.024 ; PubMed Central PMCID: PMCPMC5405160. 28365230 10.1016/j.ebiom.2017.03.024 PMC5405160

[16] Puchhammer-Stockl E, Steininger C, Geringer E, Heinz FX. Comparison of virtual phenotype and HIV-SEQ program (Stanford) interpretation for predicting drug resistance of HIV strains. HIV medicine. 2002;3(3):200–6. . 12139659 10.1046/j.1468-1293.2002.00116.x

[17] Torti C, Quiros-Roldan E, Keulen W, Scudeller L, Lo Caputo S, Boucher C, et al Comparison between rules-based human immunodeficiency virus type 1 genotype interpretations and real or virtual phenotype: concordance analysis and correlation with clinical outcome in heavily treated patients. The Journal of infectious diseases. 2003;188(2):194–201. doi: 10.1086/376512 . 12854073 10.1086/376512

[18] Gallego O, Martin-Carbonero L, Aguero J, de Mendoza C, Corral A, Soriano V. Correlation between rules-based interpretation and virtual phenotype interpretation of HIV-1 genotypes for predicting drug resistance in HIV-infected individuals. Journal of virological methods. 2004;121(1):115–8. doi: 10.1016/j.jviromet.2004.06.003 . 15350741 10.1016/j.jviromet.2004.06.003

[19] Beerenwinkel N, Daumer M, Oette M, Korn K, Hoffmann D, Kaiser R, et al Geno2pheno: Estimating phenotypic drug resistance from HIV-1 genotypes. Nucleic Acids Res. 2003;31(13):3850–5. ; PubMed Central PMCID: PMCPMC168981. 12824435 10.1093/nar/gkg575 PMC168981

[20] Vermeiren H, Van Craenenbroeck E, Alen P, Bacheler L, Picchio G, Lecocq P. Prediction of HIV-1 drug susceptibility phenotype from the viral genotype using linear regression modeling. Journal of virological methods. 2007;145(1):47–55. Epub 2007/06/19. doi: 10.1016/j.jviromet.2007.05.009 . 17574687 10.1016/j.jviromet.2007.05.009

[21] Jiamsakul A, Kantor R, Li PC, Sirivichayakul S, Sirisanthana T, Kantipong P, et al Comparison of predicted susceptibility between genotype and virtual phenotype HIV drug resistance interpretation systems among treatment-naive HIV-infected patients in Asia: TASER-M cohort analysis. BMC Res Notes. 2012;5:582 doi: 10.1186/1756-0500-5-582 ; PubMed Central PMCID: PMCPMC3505153. 23095645 10.1186/1756-0500-5-582 PMC3505153

[22] Zazzi M, Incardona F, Rosen-Zvi M, Prosperi M, Lengauer T, Altmann A, et al Predicting response to antiretroviral treatment by machine learning: the EuResist project. Intervirology. 2012;55(2):123–7. doi: 10.1159/000332008 . 22286881 10.1159/000332008

[23] Altmann A, Daumer M, Beerenwinkel N, Peres Y, Schulter E, Buch J, et al Predicting the response to combination antiretroviral therapy: retrospective validation of geno2pheno-THEO on a large clinical database. The Journal of infectious diseases. 2009;199(7):999–1006. doi: 10.1086/597305 . 19239365 10.1086/597305

[24] Revell AD, Wang D, Boyd MA, Emery S, Pozniak AL, De Wolf F, et al The development of an expert system to predict virological response to HIV therapy as part of an online treatment support tool. AIDS. 2011;25(15):1855–63. doi: 10.1097/QAD.0b013e328349a9c2 . 21785323 10.1097/QAD.0b013e328349a9c2

[25] Larder B, Wang D, Revell A, Montaner J, Harrigan R, De Wolf F, et al The development of artificial neural networks to predict virological response to combination HIV therapy. Antivir Ther. 2007;12(1):15–24. . 17503743

[26] Rhee SY, Fessel WJ, Liu TF, Marlowe NM, Rowland CM, Rode RA, et al Predictive value of HIV-1 genotypic resistance test interpretation algorithms. The Journal of infectious diseases. 2009;200(3):453–63. doi: 10.1086/600073 ; PubMed Central PMCID: PMCPMC4774893. 19552527 10.1086/600073 PMC4774893

[27] Frentz D, Boucher CA, Assel M, De Luca A, Fabbiani M, Incardona F, et al Comparison of HIV-1 genotypic resistance test interpretation systems in predicting virological outcomes over time. PloS one. 2010;5(7):e11505 doi: 10.1371/journal.pone.0011505 ; PubMed Central PMCID: PMCPMC2901338. 20634893 10.1371/journal.pone.0011505 PMC2901338

[28] Vercauteren J, Beheydt G, Prosperi M, Libin P, Imbrechts S, Camacho R, et al Clinical evaluation of Rega 8: an updated genotypic interpretation system that significantly predicts HIV-therapy response. PloS one. 2013;8(4):e61436 doi: 10.1371/journal.pone.0061436 ; PubMed Central PMCID: PMCPMC3629176. 23613852 10.1371/journal.pone.0061436 PMC3629176

[29] Sturmer M, Doerr HW, Staszewski S, Preiser W. Comparison of nine resistance interpretation systems for HIV-1 genotyping. Antivir Ther. 2003;8(3):239–44. . 12924541

[30] Poonpiriya V, Sungkanuparph S, Leechanachai P, Pasomsub E, Watitpun C, Chunhakan S, et al A study of seven rule-based algorithms for the interpretation of HIV-1 genotypic resistance data in Thailand. Journal of virological methods. 2008;151(1):79–86. doi: 10.1016/j.jviromet.2008.03.017 . 18462814 10.1016/j.jviromet.2008.03.017

[31] Vercauteren J, Vandamme AM. Algorithms for the interpretation of HIV-1 genotypic drug resistance information. Antiviral research. 2006;71(2–3):335–42. doi: 10.1016/j.antiviral.2006.05.003 . 16782210 10.1016/j.antiviral.2006.05.003

[32] Margot NA, Liu Y, Miller MD, Callebaut C. High resistance barrier to tenofovir alafenamide is driven by higher loading of tenofovir diphosphate into target cells compared to tenofovir disoproxil fumarate. Antiviral research. 2016;132:50–8. doi: 10.1016/j.antiviral.2016.05.012 . 27208653 10.1016/j.antiviral.2016.05.012

[33] Margot NA, Johnson A, Miller MD, Callebaut C. Characterization of HIV-1 Resistance to Tenofovir Alafenamide In Vitro. Antimicrobial agents and chemotherapy. 2015;59(10):5917–24. doi: 10.1128/AAC.01151-15 ; PubMed Central PMCID: PMCPMC4576099. 26149983 10.1128/AAC.01151-15 PMC4576099

[34] US Department of Health and Human Services Panel on Clinical Practices for Treatment of HIV Infection A. Guidelines for the use of antiretroviral agents in HIV-1-infected adults and adolescents (July 2016), http://aidsinfo.nih.gov/guidelines. 2016. 10.1310/hct.2000.1.1.008 11590490

[35] TenoRes Study G. Global epidemiology of drug resistance after failure of WHO recommended first-line regimens for adult HIV-1 infection: a multicentre retrospective cohort study. The Lancet infectious diseases. 2016 doi: 10.1016/S1473-3099(15)00536-8 ; PubMed Central PMCID: PMCPMC4835583. 26831472 10.1016/S1473-3099(15)00536-8 PMC4835583

[36] Whitcomb JM, Parkin NT, Chappey C, Hellmann NS, Petropoulos CJ. Broad nucleoside reverse-transcriptase inhibitor cross-resistance in human immunodeficiency virus type 1 clinical isolates. The Journal of infectious diseases. 2003;188(7):992–1000. Epub 2003/09/27. doi: 10.1086/378281 . 14513419 10.1086/378281

[37] Melikian GL, Rhee SY, Taylor J, Fessel WJ, Kaufman D, Towner W, et al Standardized comparison of the relative impacts of HIV-1 reverse transcriptase (RT) mutations on nucleoside RT inhibitor susceptibility. Antimicrobial agents and chemotherapy. 2012;56(5):2305–13. Epub 2012/02/15. doi: 10.1128/AAC.05487-11 ; PubMed Central PMCID: PMC3346663. 22330916 10.1128/AAC.05487-11 PMC3346663

[38] Miller MD, Margot N, Lu B, Zhong L, Chen SS, Cheng A, et al Genotypic and phenotypic predictors of the magnitude of response to tenofovir disoproxil fumarate treatment in antiretroviral-experienced patients. The Journal of infectious diseases. 2004;189(5):837–46. Epub 2004/02/21. doi: 10.1086/381784 . 14976601 10.1086/381784

[39] Margot NA, Wong P, Kulkarni R, White K, Porter D, Abram ME, et al Commonly Transmitted HIV-1 Drug Resistance Mutations in Reverse-Transcriptase and Protease in Antiretroviral Treatment-Naive Patients and Response to Regimens Containing Tenofovir Disoproxil Fumarate or Tenofovir Alafenamide. The Journal of infectious diseases. 2017;215(6):920–7. doi: 10.1093/infdis/jix015 . 28453836 10.1093/infdis/jix015

[40] Saag MS, Sonnerborg A, Torres RA, Lancaster D, Gazzard BG, Schooley RT, et al Antiretroviral effect and safety of abacavir alone and in combination with zidovudine in HIV-infected adults. AIDS. 1998;12:F203–F9. 9833848 10.1097/00002030-199816000-00002

[41] Louie M, Hogan C, Hurley A, Simon V, Chung C, Padte N, et al Determining the antiviral activity of tenofovir disoproxil fumarate in treatment-naive chronically HIV-1-infected individuals. AIDS. 2003;17(8):1151–6. doi: 10.1097/01.aids.0000060362.78202.97 . 12819516 10.1097/00002030-200305230-00006

[42] Sax PE, Tierney C, Collier AC, Fischl MA, Mollan K, Peeples L, et al Abacavir-lamivudine versus tenofovir-emtricitabine for initial HIV-1 therapy. N Engl J Med. 2009;361(23):2230–40. doi: 10.1056/NEJMoa0906768 ; PubMed Central PMCID: PMCPMC2800041. 19952143 10.1056/NEJMoa0906768 PMC2800041

[43] Rimsky L, Vingerhoets J, Van Eygen V, Eron J, Clotet B, Hoogstoel A, et al Genotypic and phenotypic characterization of HIV-1 isolates obtained from patients on rilpivirine therapy experiencing virologic failure in the phase 3 ECHO and THRIVE studies: 48-week analysis. J Acquir Immune Defic Syndr. 2012;59(1):39–46. Epub 2011/11/10. doi: 10.1097/QAI.0b013e31823df4da . 22067667 10.1097/QAI.0b013e31823df4da

[44] Antinori A, Zaccarelli M, Cingolani A, Forbici F, Rizzo MG, Trotta MP, et al Cross-resistance among nonnucleoside reverse transcriptase inhibitors limits recycling efavirenz after nevirapine failure. AIDS research and human retroviruses. 2002;18(12):835–8. doi: 10.1089/08892220260190308 . 12201905 10.1089/08892220260190308

[45] Walmsley SL, Kelly DV, Tseng AL, Humar A, Harrigan PR. Non-nucleoside reverse transcriptase inhibitor failure impairs HIV-RNA responses to efavirenz-containing salvage antiretroviral therapy. AIDS. 2001;15(12):1581–4. . 11504994 10.1097/00002030-200108170-00019

[46] Shulman NS, Zolopa AR, Passaro DJ, Murlidharan U, Israelski DM, Brosgart CL, et al Efavirenz- and adefovir dipivoxil-based salvage therapy in highly treatment-experienced patients: clinical and genotypic predictors of virologic response. J Acquir Immune Defic Syndr. 2000;23(3):221–6. . 10839657 10.1097/00126334-200003010-00002

[47] Mackie NE, Dunn DT, Dolling D, Garvey L, Harrison L, Fearnhill E, et al The impact of HIV-1 reverse transcriptase polymorphisms on responses to first-line nonnucleoside reverse transcriptase inhibitor-based therapy in HIV-1-infected adults. AIDS. 2013;27(14):2245–53. doi: 10.1097/QAD.0b013e3283636179 . 24157905 10.1097/QAD.0b013e3283636179

[48] King MS, Rode R, Cohen-Codar I, Calvez V, Marcelin AG, Hanna GJ, et al Predictive genotypic algorithm for virologic response to lopinavir-ritonavir in protease inhibitor-experienced patients. Antimicrobial agents and chemotherapy. 2007;51(9):3067–74. Epub 2007/06/20. doi: 10.1128/AAC.00388-07 ; PubMed Central PMCID: PMC2043245. 17576846 10.1128/AAC.00388-07 PMC2043245

[49] De Meyer S, Azijn H, Surleraux D, Jochmans D, Tahri A, Pauwels R, et al TMC114, a novel human immunodeficiency virus type 1 protease inhibitor active against protease inhibitor-resistant viruses, including a broad range of clinical isolates. Antimicrobial agents and chemotherapy. 2005;49(6):2314–21. Epub 2005/05/27. doi: 10.1128/AAC.49.6.2314-2321.2005 ; PubMed Central PMCID: PMC1140553. 15917527 10.1128/AAC.49.6.2314-2321.2005 PMC1140553

[50] de Meyer S, Vangeneugden T, van Baelen B, de Paepe E, van Marck H, Picchio G, et al Resistance profile of darunavir: combined 24-week results from the POWER trials. AIDS research and human retroviruses. 2008;24(3):379–88. Epub 2008/03/11. doi: 10.1089/aid.2007.0173 . 18327986 10.1089/aid.2007.0173

[51] Kempf DJ, Isaacson JD, King MS, Brun SC, Xu Y, Real K, et al Identification of genotypic changes in human immunodeficiency virus protease that correlate with reduced susceptibility to the protease inhibitor lopinavir among viral isolates from protease inhibitor-experienced patients. J Virol. 2001;75(16):7462–9. Epub 2001/07/20. doi: 10.1128/JVI.75.16.7462-7469.2001 ; PubMed Central PMCID: PMC114981. 11462018 10.1128/JVI.75.16.7462-7469.2001 PMC114981

[52] Rhee SY, Taylor J, Fessel WJ, Kaufman D, Towner W, Troia P, et al HIV-1 protease mutations and protease inhibitor cross-resistance. Antimicrobial agents and chemotherapy. 2010;54(10):4253–61. Epub 2010/07/28. doi: 10.1128/AAC.00574-10 ; PubMed Central PMCID: PMC2944562. 20660676 10.1128/AAC.00574-10 PMC2944562

[53] Naeger LK, Struble KA. Food and Drug Administration analysis of tipranavir clinical resistance in HIV-1-infected treatment-experienced patients. AIDS. 2007;21(2):179–85. Epub 2007/01/02. doi: 10.1097/QAD.0b013e3280119213 . 17197808 10.1097/QAD.0b013e3280119213

[54] Malet I, Delelis O, Valantin MA, Montes B, Soulie C, Wirden M, et al Mutations associated with failure of raltegravir treatment affect integrase sensitivity to the inhibitor in vitro. Antimicrobial agents and chemotherapy. 2008;52(4):1351–8. doi: 10.1128/AAC.01228-07 ; PubMed Central PMCID: PMCPMC2292515. 18227187 10.1128/AAC.01228-07 PMC2292515

[55] Molina JM, Lamarca A, Andrade-Villanueva J, Clotet B, Clumeck N, Liu YP, et al Efficacy and safety of once daily elvitegravir versus twice daily raltegravir in treatment-experienced patients with HIV-1 receiving a ritonavir-boosted protease inhibitor: randomised, double-blind, phase 3, non-inferiority study. The Lancet infectious diseases. 2012;12(1):27–35. Epub 2011/10/22. doi: 10.1016/S1473-3099(11)70249-3 . 22015077 10.1016/S1473-3099(11)70249-3

[56] Eron JJ, Clotet B, Durant J, Katlama C, Kumar P, Lazzarin A, et al Safety and Efficacy of Dolutegravir in Treatment-Experienced Subjects With Raltegravir-Resistant HIV Type 1 Infection: 24-Week Results of the VIKING Study. The Journal of infectious diseases. 2013;207(5):740–8. Epub 2012/12/12. doi: 10.1093/infdis/jis750 ; PubMed Central PMCID: PMC3563307. 23225901 10.1093/infdis/jis750 PMC3563307

[57] Danion F, Belissa E, Peytavin G, Thierry E, Lanternier F, Scemla A, et al Non-virological response to a dolutegravir-containing regimen in a patient harbouring a E157Q-mutated virus in the integrase region. The Journal of antimicrobial chemotherapy. 2015;70(6):1921–3. doi: 10.1093/jac/dkv012 . 25670643 10.1093/jac/dkv012

[58] Rhee SY, Sankaran K, Varghese V, Winters M, Hurt CB, Eron JJ, et al HIV-1 Protease, Reverse Transcriptase, and Integrase Variation. J Virol. 2016 doi: 10.1128/JVI.00495-16 . 27099321 10.1128/JVI.00495-16 PMC4907232

[59] Fransen S, Gupta S, Frantzell A, Petropoulos CJ, Huang W. Substitutions at amino acid positions 143, 148, and 155 of HIV-1 integrase define distinct genetic barriers to raltegravir resistance in vivo. J Virol. 2012;86(13):7249–55. Epub 2012/05/04. doi: 10.1128/JVI.06618-11 ; PubMed Central PMCID: PMC3416338. 22553340 10.1128/JVI.06618-11 PMC3416338

[60] Abram ME, Ram RR, Margot NA, Barnes TL, White KL, Callebaut C, et al Lack of impact of pre-existing T97A HIV-1 integrase mutation on integrase strand transfer inhibitor resistance and treatment outcome. PloS one. 2017;12(2):e0172206 doi: 10.1371/journal.pone.0172206 ; PubMed Central PMCID: PMCPMC5315389. 28212411 10.1371/journal.pone.0172206 PMC5315389

[61] Quashie PK, Mesplede T, Han YS, Oliveira M, Singhroy DN, Fujiwara T, et al Characterization of the R263K mutation in HIV-1 integrase that confers low-level resistance to the second-generation integrase strand transfer inhibitor dolutegravir. J Virol. 2012;86(5):2696–705. Epub 2011/12/30. doi: 10.1128/JVI.06591-11 ; PubMed Central PMCID: PMC3302270. 22205735 10.1128/JVI.06591-11 PMC3302270

[62] Mesplede T, Quashie PK, Osman N, Han Y, Singhroy DN, Lie Y, et al Viral fitness cost prevents HIV-1 from evading dolutegravir drug pressure. Retrovirology. 2013;10(1):22 Epub 2013/02/26. doi: 10.1186/1742-4690-10-22 . 23432922 10.1186/1742-4690-10-22 PMC3598531

[63] Cahn P, Pozniak AL, Mingrone H, Shuldyakov A, Brites C, Andrade-Villanueva JF, et al Dolutegravir versus raltegravir in antiretroviral-experienced, integrase-inhibitor-naive adults with HIV: week 48 results from the randomised, double-blind, non-inferiority SAILING study. Lancet. 2013;382(9893):700–8. doi: 10.1016/S0140-6736(13)61221-0 . 23830355 10.1016/S0140-6736(13)61221-0

[64] Lepik KJ, Harrigan PR, Yip B, Wang L, Robbins MA, Zhang WW, et al Emergent drug resistance with integrase strand transfer inhibitor-based regimens: Incidence and risk factors. AIDS. 2017 doi: 10.1097/QAD.0000000000001494 . 28375875 10.1097/QAD.0000000000001494

[65] Blanco JL, Oldenbuettel C, Thomas R, Mallolas J, Wolf E, Brenner B, et al. Pathways of resistance in subjects failing dolutegravir monotherapy (abstract 42). 2017 Conference on Retroviruses and Opportunistic Infections, Seattle WA, USA, Feb 13–16, 2017. 2017.

[66] Anstett K, Mesplede T, Oliveira M, Cutillas V, Wainberg MA. Dolutegravir resistance mutation R263K cannot coexist in combination with many classical integrase inhibitor resistance substitutions. J Virol. 2015;89(8):4681–4. doi: 10.1128/JVI.03485-14 ; PubMed Central PMCID: PMCPMC4442391. 25653436 10.1128/JVI.03485-14 PMC4442391

[67] Brenner BG, Thomas R, Blanco JL, Ibanescu RI, Oliveira M, Mesplede T, et al Development of a G118R mutation in HIV-1 integrase following a switch to dolutegravir monotherapy leading to cross-resistance to integrase inhibitors. The Journal of antimicrobial chemotherapy. 2016 doi: 10.1093/jac/dkw071 . 27029845 10.1093/jac/dkw071 PMC4896408

[68] Quashie PK, Mesplede T, Han YS, Veres T, Osman N, Hassounah S, et al Biochemical analysis of the role of G118R-linked dolutegravir drug resistance substitutions in HIV-1 integrase. Antimicrobial agents and chemotherapy. 2013;57(12):6223–35. doi: 10.1128/AAC.01835-13 ; PubMed Central PMCID: PMCPMC3837891. 24080645 10.1128/AAC.01835-13 PMC3837891

[69] Quashie PK, Oliviera M, Veres T, Osman N, Han YS, Hassounah S, et al Differential effects of the G118R, H51Y, and E138K resistance substitutions in different subtypes of HIV integrase. J Virol. 2015;89(6):3163–75. doi: 10.1128/JVI.03353-14 ; PubMed Central PMCID: PMCPMC4337543. 25552724 10.1128/JVI.03353-14 PMC4337543

[70] Malet I, Gimferrer Arriaga L, Artese A, Costa G, Parrotta L, Alcaro S, et al New raltegravir resistance pathways induce broad cross-resistance to all currently used integrase inhibitors. The Journal of antimicrobial chemotherapy. 2014;69(8):2118–22. doi: 10.1093/jac/dku095 . 24710029 10.1093/jac/dku095

[71] Munir S, Thierry E, Malet I, Subra F, Calvez V, Marcelin AG, et al G118R and F121Y mutations identified in patients failing raltegravir treatment confer dolutegravir resistance. The Journal of antimicrobial chemotherapy. 2015;70(3):739–49. doi: 10.1093/jac/dku474 . 25414202 10.1093/jac/dku474

[72] Underwood MR, Johns BA, Sato A, Martin JN, Deeks SG, Fujiwara T. The activity of the integrase inhibitor dolutegravir against HIV-1 variants isolated from raltegravir-treated adults. J Acquir Immune Defic Syndr. 2012;61(3):297–301. Epub 2012/08/11. doi: 10.1097/QAI.0b013e31826bfd02 . 22878423 10.1097/QAI.0b013e31826bfd02 PMC3804312

[73] Canducci F, Ceresola ER, Boeri E, Spagnuolo V, Cossarini F, Castagna A, et al Cross-resistance profile of the novel integrase inhibitor Dolutegravir (S/GSK1349572) using clonal viral variants selected in patients failing raltegravir. The Journal of infectious diseases. 2011;204(11):1811–5. Epub 2011/10/11. doi: 10.1093/infdis/jir636 . 21984737 10.1093/infdis/jir636

[74] World Health Organization HIV/AIDS Programme. Consolidated guidelines on the use of antiretroviral drugs for treating and preventing HIV infection. 2013. 24716260

[75] Lanier ER, Ait-Khaled M, Scott J, Stone C, Melby T, Sturge G, et al Antiviral efficacy of abacavir in antiretroviral therapy-experienced adults harbouring HIV-1 with specific patterns of resistance to nucleoside reverse transcriptase inhibitors. Antivir Ther. 2004;9(1):37–45. . 15040535 10.1177/135965350400900102

[76] Vingerhoets J, Tambuyzer L, Azijn H, Hoogstoel A, Nijs S, Peeters M, et al Resistance profile of etravirine: combined analysis of baseline genotypic and phenotypic data from the randomized, controlled Phase III clinical studies. AIDS. 2010;24(4):503–14. Epub 2010/01/07. doi: 10.1097/QAD.0b013e32833677ac . 20051805 10.1097/QAD.0b013e32833677ac

