# Supplementary material for: Collaborative update of a rule-based expert system for HIV-1 genotypic resistance test interpretation
Source: PLoS One. 2017 Jul 28;12(7):e0181357. doi: 10.1371/journal.pone.0181357 (PMC5533429; doi:10.1371/journal.pone.0181357)
Supplement: S1 File — (DOCX) [file pone.0181357.s001.docx]

**Expert panel questionnaire**

| ARV Class | ARV | Include Y/N? |
| --- | --- | --- |
| NRTIs | ABC |  |
|  | AZT |  |
|  | D4T |  |
|  | DDI |  |
|  | FTC |  |
|  | 3TC |  |
|  | TDF |  |
| NNRTIs | EFV |  |
|  | ETR |  |
|  | NVP |  |
|  | RPV |  |
| Pis | ATV |  |
|  | DRV |  |
|  | FPV |  |
|  | IDV |  |
|  | LPV |  |
|  | NFV |  |
|  | SQV |  |
|  | TPV |  |
| INSTIs | DTG |  |
|  | EVG |  |
|  | RAL |  |

|  |  |  |  | **Please rank as Susceptible, Potential Low, Low, Intermediate, High** | | | |
| --- | --- | --- | --- | --- | --- | --- | --- |
| NRTIs | Pattern |  |  | **TDF-Level** | **3FTC-Level** | **ABC-Level** | **AZT-Level** |
|  | K65R |  |  |  |  |  |  |
|  | M184V |  |  |  |  |  |  |
|  | K65R+M184V |  |  |  |  |  |  |
|  | K65N |  |  |  |  |  |  |
|  | K70EGQ |  |  |  |  |  |  |
|  | Q151M |  |  |  |  |  |  |
|  | F77L+F116Y+Q151M |  |  |  |  |  |  |
|  |  |  |  |  |  |  |  |
|  | T215FY |  |  |  |  |  |  |
|  | M41L+T215FY |  |  |  |  |  |  |
|  | M41L+M184V+T215FY |  |  |  |  |  |  |
|  | M41L+L210W+T215FY |  |  |  |  |  |  |
|  | M41L+M184V+L210W+T215FY |  |  |  |  |  |  |
|  | D67N+K70R+M184V+K219QE |  |  |  |  |  |  |
|  |  |  |  |  |  |  |  |
|  |  |  |  | **Please rank as Susceptible, Potential Low, Low, Intermediate, High** | | | |
| NNRTIs | Pattern |  |  | **EFV-Level** | **RPV-Level** | **ETR-Level** |  |
|  | E138A |  |  |  |  |  |  |
|  | E138GQ |  |  |  |  |  |  |
|  | E138K |  |  |  |  |  |  |
|  | A98G |  |  |  |  |  |  |
|  | K101E |  |  |  |  |  |  |
|  | V179D |  |  |  |  |  |  |
|  | Y181C |  |  |  |  |  |  |
|  | L100I+K103N |  |  |  |  |  |  |
|  | K101E+G190A |  |  |  |  |  |  |
|  |  |  |  |  |  |  |  |
|  |  |  |  | **Please rank as Susceptible, Potential Low, Low, Intermediate, High** | | | |
| PIs | Pattern |  |  | **ATV-Level** | **LPV-Level** | **DRV-Level** |  |
|  | M46IL |  |  |  |  |  |  |
|  | L90M |  |  |  |  |  |  |
|  | M46I+L90M |  |  |  |  |  |  |
|  | M46I+I84V+L90M |  |  |  |  |  |  |
|  | M46L+I54V+V82A |  |  |  |  |  |  |
|  |  |  |  |  |  |  |  |
|  | L76V |  |  |  |  |  |  |
|  | I50V |  |  |  |  |  |  |
|  | I54ML |  |  |  |  |  |  |
|  | V32I+I47V |  |  |  |  |  |  |
|  | V32I+L33F+M46I+I47V+I54M |  |  |  |  |  |  |
|  | L33F+M46I+I47V I54M+I84V+L90M | |  |  |  |  |  |
|  | L33F+M46L+I54M+I84V+L89V+L90M | |  |  |  |  |  |
|  | V32I+M46I+I47V I84V |  |  |  |  |  |  |
|  |  |  |  |  |  |  |  |
|  |  |  |  | **Please rank as Susceptible, Potential Low, Low, Intermediate, High** | | | |
| INSTIs | Pattern |  |  | **RAL-Level** | **EVG-Level** | **DTG-Level** |  |
|  | Q148HRK |  |  |  |  |  |  |
|  | G140SCA |  |  |  |  |  |  |
|  | E138KAT |  |  |  |  |  |  |
|  | 140SCA+148HRK |  |  |  |  |  |  |
|  | E138KAT+148HRK |  |  |  |  |  |  |
|  | E138KAT+140SAC+148HRK |  |  |  |  |  |  |
|  |  |  |  |  |  |  |  |
|  | T97A |  |  |  |  |  |  |
|  | E157Q |  |  |  |  |  |  |
|  |  |  |  |  |  |  |  |
|  | E92Q |  |  |  |  |  |  |
|  | N155H |  |  |  |  |  |  |
|  | Y143RC |  |  |  |  |  |  |
|  | T97A+Y143RC |  |  |  |  |  |  |
|  |  |  |  |  |  |  |  |
|  | R263K |  |  |  |  |  |  |
|  | G118R |  |  |  |  |  |  |
